# Trough concentration of voriconazole and its relationship with efficacy and safety: a systematic review and meta-analysis

**DOI:** 10.1093/jac/dkw045

**Published:** 2016-03-10

**Authors:** Haiying Jin, Tiansheng Wang, Bonnie A. Falcione, Keith M. Olsen, Ken Chen, Huilin Tang, John Hui, Suodi Zhai

**Affiliations:** 1 Department of Pharmacy, Peking University Third Hospital, Beijing, China; 2 Department of Pharmacy, The Affiliated Hospital of Medical College, Ningbo University, Ningbo, Zhejiang, China; 3 Department of Pharmacy Administration and Clinical Pharmacy, Peking University School of Pharmaceutical Sciences, Beijing, China; 4 Department of Pharmacy and Therapeutics, School of Pharmacy, University of Pittsburgh, Pittsburgh, PA, USA; 5 Department of Pharmacy Practice, University of Arkansas for Medical Sciences, Little Rock, AR, USA; 6 Department of Pharmacy, Stanford University Hospital and Clinics, Palo Alto, CA, USA

## Abstract

**Objectives:**

The optimum trough concentration of voriconazole for clinical response and safety is controversial. The objective of this review was to determine the optimum trough concentration of voriconazole and evaluate its relationship with efficacy and safety.

**Methods:**

MEDLINE, EMBASE, ClinicalTrials.gov, the Cochrane Library and three Chinese literature databases were searched. Observational studies that compared clinical outcomes below and above the trough concentration cut-off value were included. We set the trough concentration cut-off value for efficacy as 0.5, 1.0, 1.5, 2.0 and 3.0 mg/L and for safety as 3.0, 4.0, 5.0, 5.5 and 6.0 mg/L. The efficacy outcomes were invasive fungal infection-related mortality, all-cause mortality, rate of successful treatment and rate of prophylaxis failure. The safety outcomes included incidents of hepatotoxicity, neurotoxicity and visual disorders.

**Results:**

A total of 21 studies involving 1158 patients were included. Compared with voriconazole trough concentrations of >0.5 mg/L, levels of <0.5 mg/L significantly decreased the rate of treatment success (risk ratio = 0.46, 95% CI 0.29–0.74). The incidence of hepatotoxicity was significantly increased with trough concentrations >3.0, >4.0, >5.5 and >6.0 mg/L. The incidence of neurotoxicity was significantly increased with trough concentrations >4.0 and >5.5 mg/L.

**Conclusions:**

A voriconazole level of 0.5 mg/L should be considered the lower threshold associated with efficacy. A trough concentration >3.0 mg/L is associated with increased hepatotoxicity, particularly for the Asian population, and >4.0 mg/L is associated with increased neurotoxicity.

## Abstract (precis)

This meta-analysis showed trough concentrations of 0.5 mg/L to be the lower limit of voriconazole during treatment, whereas trough concentrations of 3.0 mg/L were associated with an increased risk of moderate to severe hepatotoxicity, particularly for the Asian population.

## Introduction

Deep mycoses are serious infections associated with a high mortality. In 77% of patients with invasive fungal infection (IFI), their IFI were significantly related to their death.^1^ Voriconazole is a second-generation triazole antifungal agent with a broad spectrum of activity, which is often recommended as primary therapy for IFI^2–6^ and as antifungal prophylaxis in immunocompromised patients.^7^ To improve treatment outcomes of voriconazole, therapeutic drug monitoring (TDM) is suggested in major guidelines from the IDSA, the American Thoracic Society and ESCMID.^2–6^ Voriconazole trough concentrations are good measures of drug exposure,^8^ but the aforementioned guidelines do not explicitly recommend an optimum trough concentration.

To our knowledge, no randomized trials have evaluated the target trough concentration of voriconazole in deep mycoses. However, numerous observational studies have recommended lowest voriconazole concentration cut-off values, including 0.25,^9^ 1,^10^ 1.2,^11^ 1.5,^12^ 1.7,^13^ 2^14^ and 2.2 mg/L.^15^ A guideline authored by two Japanese societies and published in 2013 recommended a voriconazole target trough concentration of 1–2 mg/L for efficacy and a trough concentration >4–5 mg/L as a critical concentration for potentially attributable elevated liver function tests,^16^ which was primarily based on a meta-analysis of observational studies by Hamada *et al*.^17^ In 2014 the British Society for Medical Mycology recommended a trough concentration >1 mg/L or a trough/MIC ratio of 2–5 as a target for efficacy and a trough concentration of <4–6 mg/L for safety,^18^ which was based on a large observational study by Troke,^22^ smaller observational studies^9,10,13,14,19–22^ and the previous meta-analysis.^17^

However, the evidence supporting the voriconazole target and critical trough concentrations described in these two guidelines has important limitations. For example, Troke *et al*. used simulation data derived from a Monte Carlo model rather than actual patient data.^22^ Furthermore, the previous meta-analysis^17^ has drawbacks such as a lack of inclusion of eligible studies (searched only PubMed from its inception until April 2009), a lack of standardization for outcome definitions among included studies, including a study^23^ that evaluated voriconazole random concentration rather than trough concentration, and inadequate subgroup analysis to explore the heterogeneity. Therefore, it is necessary to perform an updated meta-analysis to provide recommendations for the optimum voriconazole trough concentration. The objective of this study was to evaluate the relationship between the reported voriconazole trough concentration, and efficacy and safety of voriconazole in patients with, or at risk for, deep mycoses.

## Methods

We followed the methods specified in the Cochrane Handbook for Systematic Reviews^24^ and the Meta-analysis of Observational Studies in Epidemiology guidelines.^25^

### Data sources

Eligible trials were identified through electronic and manual searches. Electronic searches were performed in MEDLINE, EMBASE, Cochrane Library, ClinicalTrials.gov and three Chinese literature databases (CNKI, WanFang, CBM) from their inception until March 2015. The search was limited to English or Chinese articles. We used the keyword ‘voriconazole’ to search these databases. Manual searches included scanning of reference lists in relevant papers.

### Study selection

Initial screening was conducted by a group of clinical pharmacists. Two reviewers (H. J., K. C.) independently assessed titles, abstracts and citations in greater detail. Studies were included if: (i) observational study; (ii) voriconazole was used for treatment or prophylaxis; (iii) TDM was performed; (iv) trough concentrations at steady state were reported for included patients; (v) rate of treatment success, rate of prophylaxis failure, mortality or incidence of voriconazole-related adverse events (hepatotoxicity, neurotoxicity, visual disorder) at both below and above the cut-off value of the trough concentration were reported for included patients, or sufficient data to estimate these was provided; (vi) sample size was ≥10 patients; and (vii) full text of the publication was available. Full text of potentially relevant articles was retrieved and assessed by the same reviewers using the criteria above. Disagreements were resolved through discussion.

Our exclusion criteria included: (i) data came from simulated patients or pharmacokinetic models rather than from real patients; (ii) concentrations were not troughs; or (iii) concentrations were not measured at steady state.

### Outcome measures

The efficacy outcomes included were: IFI-related death; all-cause mortality; treatment success; and prophylaxis failure. Given the known variation in the definitions of treatment success in the literature, we used the criteria from the majority of included studies to minimize heterogeneity (complete and partial response). Definitions of outcomes are provided in Table S1 (available as Supplementary data at *JAC* Online). Prophylaxis failure was evaluated by the incidence of IFIs; a high risk ratio (RR) meant a high prophylaxis failure rate. The safety outcomes were hepatotoxicity, neurotoxicity and visual disorders. The pooled analysis for treatment success included only treatment studies, for prophylaxis failure only prophylaxis studies and analysis of side effects included all studies.

### Cut-off value establishment

According to previous studies,^10,14,26–28^ the MIC_90_ (MIC at which 90% of isolates were inhibited) of voriconazole for most yeasts and moulds is between 0.5 and 1 mg/L,^26–28^ and patients with voriconazole trough concentrations >2 mg/L were associated with good clinical response.^14^ Some studies have shown that the most likely target concentration for efficacy is >1 mg/L^10,29^ and one study recommended 1.5 mg/L as the target concentration.^12^ Thus we established the stepwise cut-off values for efficacy between 0.5 and 3.0 mg/L (0.5, 1.0, 1.5, 2.0 and 3.0 mg/L).

A target voriconazole trough concentration <4–6 mg/L was suggested by the British Society for Medical Mycology to minimize drug-related toxicity.^18^ Previous studies^10,30,31^ have evaluated 5.5 mg/L as a cut-off concentration for toxicity. Thus, we set the stepwise cut-off values for voriconazole safety between 3.0 and 6.0 mg/L (3.0, 4.0, 5.0, 5.5, 6.0 mg/L).

### Data extraction

Two authors extracted data independently (H. J. and K. C.) and disagreements were resolved by discussion or by a third investigator (T. W.). From each study, we extracted study characteristics, participants' baseline characteristics, methods for measuring voriconazole trough concentration, type of trough concentration (initial, mean or maximum), cut-off value of voriconazole trough concentration and pre-specified study outcomes of efficacy and safety.

As our outcomes were all dichotomous, we used the number of events (numerator) and sample size (denominator) to perform the meta-analysis. For each study, we considered patient groups treated with voriconazole at a concentration below the pre-defined cut-off value as the intervention group, and patient groups treated with voriconazole at a concentration above the pre-defined cut-off value as the control. When individual patient data were available, we used all of our pre-defined cut-off values to divide patients into two groups in the same way and extracted the number of events.

For efficacy, when the trough concentration was measured multiple times for each patient, we used the mean value of multiple measurements for that patient; median value was used only when the mean was not available. For safety, we extracted the highest trough concentration for each patient; if it was not available, we used the reported trough concentration for that patient in the article. If there were multiple data for the same outcome in an article, only outcome data with the longest follow-up were extracted. According to a previous method,^32^ if concentration values were below the detection limit for a certain value, we set the concentration as half of this value (e.g. individual data provided by Kim *et al*.^29^ showed trough concentrations <0.5 mg/L in some cases, so we defined these trough concentrations as 0.25 mg/L). When necessary, we contacted the study's corresponding author for clarification, or requested additional data.

### Quality assessment

The Newcastle–Ottawa Scale was applied to evaluate the quality of the included studies.^33^ This scale uses a star system (maximum of nine stars) to evaluate the methodological quality of each study.

### Data analysis

Meta-analysis and assessment of publication bias was performed using RevMan 5.1 (Cochran IMS) and Stata version 12.0 (StataCorp LP). To assess variations between studies in addition to sampling error within studies, the random-effects model was selected. The Mantel–Haenszel method was used to calculate the RR and 95% CI for each study. The Cochran Q χ^2^ test and *I*^2^ statistic were used to assess heterogeneity among studies. *I*^2^ values of over 25%, 50% and 75% represent low, moderate and considerable heterogeneity, respectively.^24^ *P* < 0.05 was considered statistically significant.

To explore the heterogeneity among different studies, subgroup analysis was performed when more than two studies were included in the analysis of each cut-off level. For the efficacy outcome, studies were stratified by: (i) studies exclusively including patients with proven or probable IFI compared with studies including patients with possible IFI or the category of IFI was not clearly reported; (ii) studies reporting single drug therapy compared with studies including patients on combo therapy (at least some patients on combo therapy) (since voriconazole monotherapy was recommended by the IDSA, the American Thoracic Society and ESCMID,^2–6^ if a study did not report whether voriconazole was used in combination with other antifungal agents, we considered it as a monotherapy study, as long as the site of infection for the study did not include the CNS); and (iii) studies for adults compared with studies for children.

For the safety outcome, studies were stratified by (i) studies for adults compared with studies for children, and (ii) study location in Asian countries compared with study location in non-Asian countries. Previous study for genotyping of CYP2C19 showed that about 12%–23% of the Asian population could be poor metabolizers of voriconazole,^16^ which may influence the incidence of adverse effects. However, as we only evaluated concentration at steady state, CYP2C19 polymorphism would not influence our assessment; therefore, it was not considered in our subgroup analysis.

Sensitivity analysis was performed to examine whether a single study had a substantial influence on the main results. We excluded each study and evaluated its effect on the summary estimates and heterogeneity of the main analysis. We further evaluated the rate of treatment success for voriconazole monotherapy. For studies that included patients on concomitant antifungals, we extracted data from patients on monotherapy only when individual patient data were available, and excluded the study otherwise. The results for sensitivity analysis were reported if the conclusions differed. If more than 10 studies were included in the analysis of each cut-off level, publication bias was evaluated using Begg's test and Egger's weighted regression statistics.^24^

## Results

### Literature searches and study inclusion

**Figure 1.**
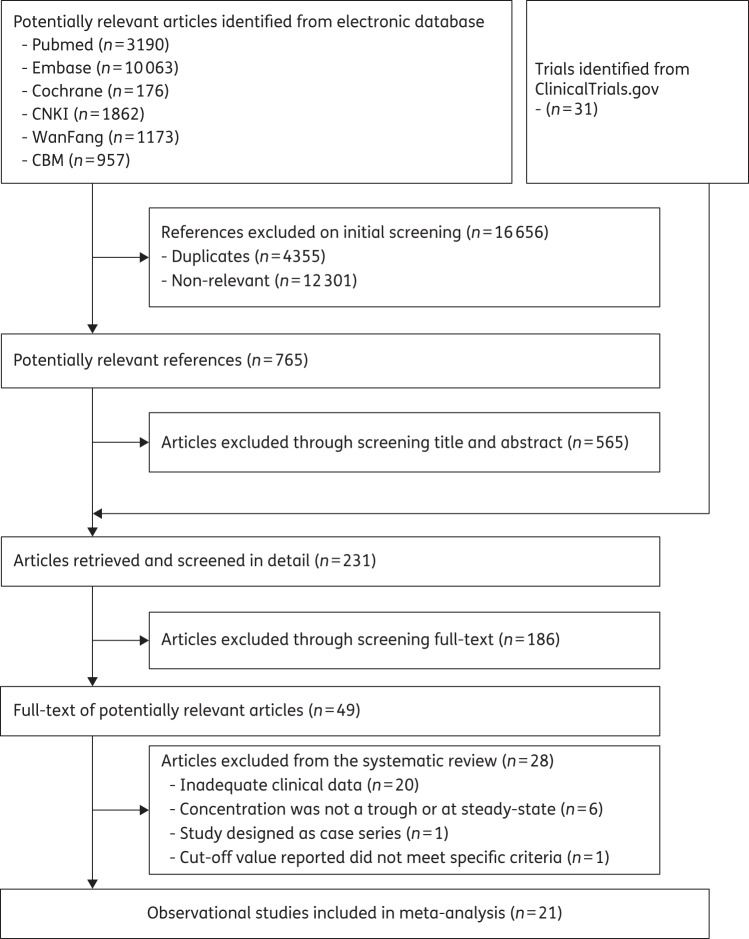
Flow chart of study selection.

The study selection process for inclusion is shown in Figure 1. The electronic searches identified 17 452 articles. After initial screening, 49 full-text, potentially relevant, articles were selected, 28 studies were excluded (the reasons for excluding are shown in Table S2) and 21 articles involving 1158 patients were included for meta-analysis.^9–12,14,19,20,29–31,34–44^ We obtained additional data from three authors.^12,14,29^

### Study descriptions

A summary of descriptions of included studies is reported in Table 1. Of these 21 studies, 9 were conducted in Asia,^11,12,14,29,30,39,41–43^ 4 included only patients diagnosed with proven or probable IFI^12,19,20,29^ and 6 included patients with concomitant use of other antifungals.^9,19,20,31,35,42^ Five studies used voriconazole for prophylaxis^34,36,40,41,44^ and 16 used voriconazole for treatment.^9–12,14,19,20,29–31,35,37–39,42,43^ Two studies were conducted in children, one used voriconazole for treatment^35^ and the other for prophylaxis.^41^ Five studies used serum samples,^19,29,30,37,38^ 13 used plasma samples and the remainder did not report whether serum or plasma sample was used.^31,39,42^ All the included studies measured voriconazole concentrations by HPLC except the study by Lee *et al*.,^39^ which used tandem MS.^41^

**Table 1.** Characteristics of included studies

Voriconazole used for treatment								
First author year	Country, study design	Sample size (male/female)	Age (years)	Main underlying disease (%)	Type of fungal infection (%)	Main site of infection (%)	Treatment duration: (days)	Combo therapy
^a^Brüggemann 2011	Netherlands, retrospective study	18 (8/10)	median: 7 IQR: 2.75–15	haematological disorder	proven (28) probable (17) possible (22) PFN (28)	lung (44) CNS (17)	NR	yes
^b^Chu 2013	USA, retrospective study	108 (59/49)	median: 53 IQR: 38–64	haematological disorder (78)	proven (7) probable (36) possible (40) PFN (8)	lung (76.8) CNS (4.6)	median: 35 range: 13–19	yes
^c^Denning 2002	UK, prospective study	122	median: 52 range: 18–79	haematological disorder, HSCT	proven (39) probable (56) possible (5)	lung CNS (16)	range: 6–168	yes
Kim 2011	Korea, prospective study	25 (12/13)	median: 45 range: 38–54	acute leukaemia (80)	NR	NR	median: 8 range: 7–14	no
^d,e^Kim 2013	Korea, prospective study	104 (54/50)	mean ± SD: 53 ± 13	haematological disorder, neutropenia (82)	proven (5) probable (95)	lung (85)	median: 116 IQR: 58–191	no
^f^Koselke 2012	USA, retrospective study	108 (63/45)	mean ± SD: 55.5 ± 14.28	haematological disorder (55) transplant (27)	NR	NR	NR	no
Lee 2013	Korea, retrospective study	52 (33/19)	range: 16–81	AML (60)	proven (4) probable (56) possible (40)	lung (90)	range: 23–131	no
^g^Okuda 2008	Japan, retrospective study	23 (11/12)	median: 64 range: 18–85	haematological disorder	proven or probable (65), PFN (26)	lung (39) uncertain (52)	NR	yes
Pascual 2008	Switzerland, retrospective study	52 (38/14)	median: 58.5 range: 23–78	neutropenia (61)	proven or probable (69) possible (21) PFN (10)	lung (58)	median: 50 range: 4–1130	no
^h^Racil 2012	Czech Republic, retrospective study	53	NR	NR	proven (21) probable (79)	NR	median: 32 range: 5–160	yes
Suzuki 2013	Japan, retrospective study	39 (18/21)	range: 12–84	NR	NR	NR	mean: 58.4 range: 7–90	no
^e^Wang 2014	China, retrospective study	144 (97/47)	median: 60.6 range: 18–99	bronchitis (24) asthma (19) liver disease (22) haematological malignancy (15)	proven (61) probable (39)	lung (76)	mean: 35.34 median: 35 range: 11–81	no
Imhof 2006	Switzerland, retrospective study	26 (19/7)	median: 47.5 range: 22–61	AML (89)	proven (27), probable (19) possible (54)	NR	NR	no
^e^Ueda 2009	Japan, retrospective study	34 (22/12)	median: 57.5 range: 19–81	haematological disorders neutropenic (47)	proven (2) probable (10) possible (59) PFN (20)	lung (65)	NR	no
^i^Gomez 2012	Spain, retrospective study	14 (10/4)	mean: 46.8 median: 54.5 range: 4–87	MHD (43) SOT (29) COPD (14)	proven (26) probable (64)	lung (50)	mean: 107 range: 9–602	yes
Matsumoto 2009	Japan, retrospective study	29 (18/11)	mean ± SD: 57.3 ± 19.3	NR	NR	NR	NR	no
Voriconazole used for prophylaxis								
First author year	Country, study design	Sample size (male/female)	Age (years)	Main disease (n.%)	Duration (days)		Follow-up (days)	
Brüggemann 2010	Netherlands, Phase 2 open-label	10 (7/3)	median: 49 range: 28–60	HSCT	14		28	
Mitsani 2012	USA, prospective study	93 (54/39)	median: 60 range: 20–74	lung transplantation (100)	≥120		NR	
Trifilio 2007	USA, retrospective study	71 (40/31)	adult	HSCT	mean: 194 range: 12–956		NR	
Heng 2013	Australia, prospective study	12 (3/9)	median: 56 range: 41–73	lung transplantation (100)	range: 11–1080		90	
^j^Mori 2015	Japan, Phase 2 open-label	21 (9/12)	range: 2–15	ALL (38) AML (33)	range: 13–21		mean ± SD: 30 ± 7	

NR, not reported; SOT, solid organ transplantation; PFN, persistent febrile neutropenia; MHD, malignant haematological disease.

^a^Thirteen patients were considered assessable for efficacy (3 patients' diagnosis of fungal infection became unlikely, and 2 patients' responses were unavailable) and 18 for safety. Three patients received combination therapy (two of them received amphotericin B and the third received caspofungin).

^b^Forty-six patients with proven and probable IFI were considered assessable for efficacy and 108 patients for safety, 9 (8.3%) patients received additional antifungal therapy with micafungin, caspofungin and/or amphotericin B.

^c^Twenty-nine (25%) patients had received amphotericin B (*n* = 21), itraconazole (*n* = 6) or amphotericin B liposomal (*n* = 2).

^d^Concentration >10 mg/L set as 10 mg/L.

^e^Obtained additional data from author.

^f^Eighty-seven patients were considered assessable for hepatotoxicity and 108 for neurotoxicity.

^g^Two patients who used voriconazole for prophylaxis assessable for efficacy. Seven patients on concomitant antifungals were excluded when sensitivity analysis was performed. Eight patients received additional antifungal therapy with amphotericin B and/or itraconazole and/or micafungin.

^h^The subgroup diagnosed as proven or probable invasive aspergillosis was used. Thirty-three patients (62%) received combined antifungal therapy with an echinocandin.

^i^Eight patients received combination therapy, and most were treated with voriconazole and caspofungin—these patients were excluded when sensitivity analysis was performed; four children were excluded when subgroup analysis was performed, which divided the adult group and the children group.

^j^Mild liver function test abnormalities were not considered as hepatotoxicity.

### Evaluation of efficacy

A summary of outcomes for each study is shown in Table 2. Summaries of meta-analysis and subgroup analysis for efficacy are shown in Tables 3–5, forest plots are shown in Figure 2 and Figures S1–19, raw data are shown in Tables S3–S6.

**Table 2.** Outcomes and results of included studies

Voriconazole used for treatment					
First author year	Type of *C*_trough_	Cut-off value	Reported outcome	Definition of treatment success	Definition of hepatotoxicity
^a,b^Brüggemann 2011	highest or mean	all	treatment success hepatotoxicity	complete, partial and stable response	NR
^c^Chu 2013	initial	1, 5.5	treatment success hepatotoxicity neurotoxicity visual disorders	complete, partial response	AST/ALT >5 × ULN or ALP/TBIL >3 × ULN
^b^Denning 2002	mean	0.5, 6.0	treatment success hepatotoxicity	complete, partial and stable response	transaminases >5 × ULN, bilirubin >3 × ULN, ALP >3 × ULN
Kim 2011	NR	6.0	hepatotoxicity neurotoxicity	—	CTCAE, grades 3–5 are referred to as SAEs
^d,e^Kim 2013	mean	all	IFI-related mortality all-cause mortality treatment success	complete or partial response	—
^f^Koselke 2012	mean	5.5	hepatotoxicity neurotoxicity	—	AST or ALT >5 × ULN
Lee 2013	initial	0.5, 1.0, 2.0, 3.0	treatment success	complete or partial response	—
^g^Okuda 2008	mean or highest	all	treatment success hepatotoxicity neurotoxicity	value of β-d-glucan improved by 50% or more	any deviation in the serological test values from the normal range or if there was no change in the assessment of these values
Psacual 2008	NR	1, 5.5	treatment success hepatotoxicity neurotoxicity	complete or partial response	CTCAE, severe cholestatic hepatopathy (defined as 10 times the baseline or 3 times the baseline, if the baseline was 13 times ULN)
^h^Racil 2012	mean	1.0, 2.0	treatment success	complete and partial response	—
Suzuki 2013	initial	4.0	hepatotoxicity	—	CTCAE, grades 2–4 after initiation of administration
^e^Wang 2014	mean	all	treatment success hepatotoxicity	complete, partial response	CTCAE, grades 3–4 AST, ALT, ALP >5 × ULN or TBIL >3 × ULN
Imhof 2006	highest	3.0, 4.0	neurotoxicity	—	NR
^e^Ueda 2009	NR	all	treatment success hepatotoxicity	complete, partial and stable response	AST, ALT, GGT or BIL was in grades 2–4 according to NCI criteria
^i^Gomez 2012	median	all	treatment success all-cause mortality	complete and partial response	—
Matsumoto 2009	only once	4.0	hepatotoxicity	—	AST, ALT, GGT or BIL was in grades 1–3 according to NCI criteria
Voriconazole used for prophylaxis					
First author year	Type of *C*_trough_	Cut-off value	Reported outcome	Definition of occurrence of IFI	Definition of hepatotoxicity
Brüggemann 2010	mean	all	visual disorders occurrence of IFI	EORTC/MSG, tracheobronchitis, positive cultures	NR
Mitsani 2012	initial	1.0, 1.5	occurrence of IFI	EORTC/MS, positive cultures	—
Trifilio 2007	NR	0.5, 1.0, 2.0, 5.0	IFI-related mortality occurrence of IFI	EORTC/MSG. Include proven, probable and possible	—
Heng 2013	mean	all	occurrence of IFI	breakthrough IFI, positive cultures	—
^j^Mori 2015	mean	all	hepatotoxicity visual disorders	—	severely: ≥2ULN at baseline, ≥5ULN on day 10; moderately: ≥2 ULN at baseline, ≥5ULN on day 7

*C*_trough_, trough concentration; NR, not reported; EORTC-MSG, European Organization for Research and Treatment of Cancer/Invasive Fungal Infections Cooperative Group and the National Institute of Allergy and Infectious Diseases Mycoses Study Group; CTCAE, Common Terminology Criteria for Adverse Events; NCI, National Cancer Institute; ULN, upper limit of normal; ALP, alkaline phosphatase; TBIL, total bilirubin; SAEs, severe adverse events.

The majority of included studies (*n* = 7) defined treatment success as complete or partial response, three studies^9,14,35^ defined treatment success as complete, partial and stable response, and a fourth^42^ defined treatment success if the β-d-glucan value improved by 50% or more. For the studies by Brüggemann *et al*. and Denning *et al*.,^9,35^ we extracted data only from the complete and partial response groups.

Nine studies defined hepatotoxicity as liver enzymes elevated >3 times upper limit of normal or above grade 2 according to the criteria of CTCAE or NCI.^9,10,12,14,29–31,41,43^ For the remaining three studies, the study by Mori *et al*. defined hepatotoxicity as any deviation in the serological test values from the normal range,^41^ we excluded the patients with mild liver dysfunction to minimize heterogeneity since the individual data were available. The study by Matsumoto *et al*. defined hepatotoxicity as absolute liver enzyme elevated within grades 1–3 according to the NCI criteria^11^ and the study by Brüggemann *et al*. did not report the definition of hepatotoxicity.^35^

^a^Thirteen patients were considered assessable for efficacy (3 patients were not included because the diagnosis of fungal infection became unlikely, and 2 patients because response was unavailable) and 18 for safety.

^b^Only data for patients with complete and partial response were extracted.

^c^Forty-six patients with proven and probable IFI were considered assessable for efficacy and 108 patients for safety.

^d^Concentration above 10 mg/L set as 10 mg/L.

^e^Obtained additional data from author.

^f^Eighty-seven patients were considered assessable for hepatotoxicity and 108 for neurotoxicity.

^g^Two patients who used voriconazole for prophylaxis were not considered assessable for efficacy; seven patients on concomitant antifungals were excluded when sensitivity analysis was performed.

^h^The subgroup diagnosed as proven or probable invasive aspergillosis was used.

^i^Eight patients who concomitantly used other antifungals were excluded when sensitivity analysis was performed; four children were excluded when subgroup analysis was performed, which separated the adult group from the paediatric group.

^j^Mild abnormal liver function was not considered as hepatotoxicity.

**Table 3.** Summary of meta-analyses for efficacy

Cut-off value (mg/L)	RR (95% CI)	No. of studies	No. of participants in experimental group	No. of participants in control group	*I*^2^ %	*P*
Rate of treatment success						
≤0.5 versus >0.5	0.46 (0.29, 0.74)	7	41	450	0	0.001
≤1.0 versus >1.0	0.88 (0.61, 1.26)	10	119	414	73	0.48
≤1.5 versus >1.5	0.93 (0.67, 1.30)	6	120	210	68	0.68
≤2.0 versus >2.0	1.01 (0.78, 1.30)	8	204	231	62	0.94
≤3.0 versus >3.0	1.03 (0.75, 1.42)	7	241	141	62	0.86
Incidence of IFI						
≤0.5 versus >0.5	1.74 (0.70, 4.31)	3	20	73	0	0.24
≤1.0 versus >1.0	1.49 (0.73, 3.01)	4	72	114	0	0.27
≤1.5 versus >1.5	1.55 (0.62, 3.84)	3	50	65	0	0.35
≤2.0 versus >2.0	0.88 (0.26, 2.95)	3	57	36	35	0.83
≤3.0 versus >3.0	0.38 (0.10, 1.38)	2	18	4	0	0.14
All-cause mortality						
≤0.5 versus >0.5	2.87 (0.32, 25.52)	2	6	112	47	0.34
≤1.0 versus >1.0	1.10 (0.16, 7.68)	2	18	100	49	0.92
≤1.5 versus >1.5	0.64 (0.13, 3.06)	2	34	84	43	0.57
≤2.0 versus >2.0	0.75 (0.13, 4.27)	2	48	70	44	0.74
≤3.0 versus >3.0	0.44 (0.22, 0.91)	2	85	33	0	0.03

**Table 4.** Summary of subgroup analyses for treatment success

Subgroup		Cut-off value (mg/L)	RR (95% CI)	No. of studies	No. of participants in experimental group	No. of participants in control group	*I*^2^ %	*P*
Category of IFI	proven + probable 100%	≤0.5 versus >0.5	0.37 (0.19, 0.72)	3	21	241	0	0.003
		≤1.0 versus >1.0	0.91 (0.55, 1.52)	4	77	238	87	0.73
		≤1.5 versus >1.5	0.92 (0.60, 1.43)	3	102	160	85	0.72
		≤2.0 versus >2.0	0.99 (0.71, 1.38)	4	168	147	80	0.95
		≤3.0 versus >3.0	1.02 (0.61, 1.70)	3	189	73	83	0.94
	proven + probable <100%	≤0.5 versus >0.5	0.58 (0.30, 1.15)	4	20	209	0	0.12
		≤1.0 versus >1.0	0.82 (0.46, 1.45)	6	42	176	48	0.49
		≤1.5 versus >1.5	0.94 (0.50, 1.76)	3	18	50	20	0.85
		≤2.0 versus >2.0	1.05 (0.64, 1.74)	4	36	84	32	0.84
		≤3.0 versus >3.0	1.02 (0.55, 1.89)	4	52	68	50	0.95
Combo therapy	yes	≤0.5 versus >0.5	0.47 (0.21, 1.05)	3	18	139	0	0.07
		≤1.0 versus >1.0	1.14 (0.70, 1.86)	5	44	103	49	0.60
		≤1.5 versus >1.5	0.86 (0.42, 1.77)	3	20	28	31	0.69
		≤2.0 versus >2.0	1.01 (0.54, 1.88)	4	62	39	57	0.97
		≤3.0 versus >3.0	0.89 (0.29, 2.69)	3	31	17	50	0.83
	no	≤0.5 versus >0.5	0.46 (0.26, 0.82)	4	23	311	0	0.009
		≤1.0 versus >1.0	0.74 (0.49, 1.14)	5	75	311	70	0.17
		≤1.5 versus >1.5	0.95 (0.62, 1.45)	3	100	182	85	0.82
		≤2.0 versus >2.0	1.00 (0.74, 1.35)	4	142	192	72	0.99
		≤3.0 versus >3.0	1.04 (0.72, 1.51)	4	210	124	77	0.83
Population	children	≤0.5 versus >0.5	NA	NA	NA	NA	NA	NA
		≤1.0 versus >1.0	1.13 (0.56, 2.25)	1	4	12	NA	0.74
		≤1.5 versus >1.5	1.35 (0.63, 3.04)	1	4	9	NA	0.47
		≤2.0 versus >2.0	1.75 (0.83, 3.67)	1	6	7	NA	0.16
		≤3.0 versus >3.0	1.60 (0.68, 3.77)	1	8	5	NA	0.28
	adults	≤0.5 versus >0.5	0.49 (0.31, 0.79)	7	39	448	0	0.003
		≤1.0 versus >1.0	0.90 (0.62, 1.31)	9	114	402	75	0.58
		≤1.5 versus >1.5	0.94 (0.65, 1.35)	5	113	200	72	0.73
		≤2.0 versus >2.0	0.99 (0.78, 1.27)	7	195	223	59	0.97
		≤3.0 versus >3.0	1.00 (0.72, 1.38)	6	237	141	63	0.98

NA, not applicable.

**Table 5.** Summary of sensitivity analyses after removing studies with combination antifungal therapy

Cut-off value (mg/L)	RR (95% CI)	No. of studies	No. of participants in experimental group	No. of participants in control group	*I*^2^ %	*P*
≤0.5 versus >0.5	0.49 (0.29, 0.81)	6	26	328	0	0.006
≤1.0 versus >1.0	0.74 (0.53, 1.03)	8	83	333	49	0.07
≤1.5 versus >1.5	0.94 (0.68, 1.30)	6	112	203	65	0.70
≤2.0 versus >2.0	0.99 (0.77, 1.29)	7	157	210	55	0.96
≤3.0 versus >3.0	1.05 (0.77, 1.44)	7	228	137	61	0.77

**Figure 2.**
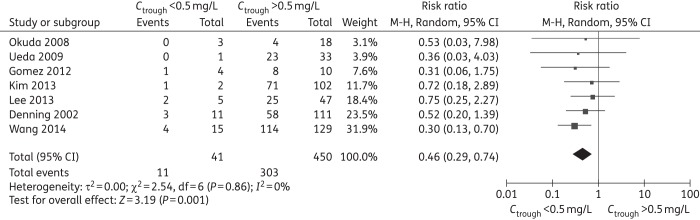
Meta-analysis for successful treatment rate (trough concentration of <0.5 mg/L comparison with >0.5 mg/L, RR <1 favours *C*_trough_ >0.5 mg/L).

There was a significant difference only at the cut-off level of 0.5 mg/L (RR = 0.46, 95% CI 0.29–0.74) (Figure 2 and Table 3). Subgroup analysis showed that rate of treatment success significantly decreased at a cut-off level of <0.5 mg/L in the following subgroups: patients with proven or probable IFI (RR = 0.37, 95% CI 0.19–0.72), monotherapy (RR = 0.46, 95% CI 0.25–0.82) and adults (RR = 0.46, 95% CI 0.26–0.82) (Table 4). There were no significant differences at other cut-off levels.

**Figure 3.**
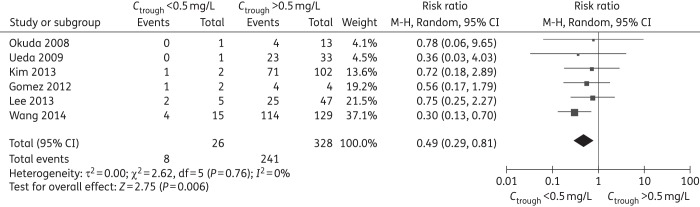
Sensitivity analysis that included only patients on monotherapy for treatment success rate (trough concentration of <0.5 mg/L comparison with >0.5 mg/L, RR <1 favours *C*_trough_ >0.5 mg/L).

The results from the sensitivity analysis including studies on concomitant antifungals, but with individual patient data on voriconazole monotherapy, showed voriconazole trough concentrations of <0.5 mg/L with a significantly lower rate of treatment success (RR = 0.49, 95% CI 0.29–0.81) (Figure 3), which further confirmed the result of subgroup analysis. There were no significant differences at other cut-off levels (Table 5 and Figures S20–23).

**Figure 4.**
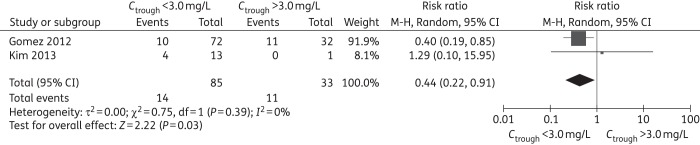
Meta-analysis for all-cause mortality (trough concentration of <3.0 mg/L comparison with >3.0 mg/L, RR <1 favours *C*_trough_ <3.0 mg/L).

Although two studies contributed data for IFI-related mortality, one study^44^ evaluated IFI prophylaxis, the other evaluated IFI treatment,^29^ thus we were unable to pool data. For all-cause mortality, our meta-analysis based on two studies showed that the rate of death significantly decreased at a cut-off level of <3.0 mg/L (RR = 0.44, 95% CI 0.22–0.91). There were no significant differences at other cut-off levels (Table 3, Figure 4 and Figures S24–27). For prophylaxis failure, the meta-analysis showed that the occurrence of IFI for voriconazole trough concentrations below the cut-off value were not significantly different from those above the same value for each evaluated cut-off level (Table 3 and Figures S28–32).

Sensitivity analysis on each study's effect on the summary estimates showed that exclusion of the study by Kim *et al*.^29^ resulted in a significantly increased rate of treatment success at trough concentrations >1.5 mg/L (Table S7).

### Evaluation of safety

Summary of primary and subgroup analysis for safety are shown in Tables 6 and 7; forest plots are shown in Figures 5 and 6 and Figures S33–55; raw data are shown in Tables S8–S10. For hepatotoxicity, the definitions varied across the 12 studies (Table 2). Our meta-analysis demonstrated a significantly lower incidence with trough concentration below cut-off levels of 3.0, 4.0, 5.5 and 6 mg/L compared with controls (Table 6 and Figure 5). Subgroup analysis showed that there were significant differences in the Asian study locations at all cut-off levels and for the adult population at cut-off levels of 3.0, 4.0, 5.5 and 6 mg/L. There was no significant difference in non-Asian study locations or paediatric populations at all cut-off levels (Table 7).

**Table 6.** Summary of meta-analyses for incidence of adverse events

Cut-off value (mg/L)	RR (95% CI)	No. of studies	No. of participants in experimental group	No. of participants in control group	*I*^2^ %	*P*
Hepatotoxicity						
≤3.0 versus >3.0	0.37 (0.16, 0.83)	5	150	90	40	0.02
≤4.0 versus >4.0	0.32 (0.14, 0.74)	7	225	83	64	0.007
≤5.0 versus >5.0	0.40 (0.16, 1.03)	5	203	37	69	0.06
≤5.5 versus >5.5	0.44 (0.28, 0.70)	8	396	91	16	<0.001
≤6.0 versus >6.0	0.41 (0.28, 0.62)	7	336	51	0	<0.001
Neurotoxicity						
≤3.0 versus >3.0	0.52 (0.13, 2.01)	2	24	25	0	0.34
≤4.0 versus >4.0	0.20 (0.05, 0.74)	2	32	17	0	0.02
≤5.0 versus >5.0	0.19 (0.01, 4.14)	1	15	8	NA	0.29
≤5.5 versus >5.5	0.37 (0.21, 0.65)	4	223	68	1	<0.001
≤6.0 versus >6.0	0.40 (0.05, 3.57)	2	35	13	0	0.41
Visual disorder						
≤3.0 versus >3.0	1.64 (0.54, 5.01)	2	24	7	0	0.38
≤4.0 versus >4.0	3.88 (0.64, 23.32)	2	26	5	0	0.14
≤5.0 versus >5.0	2.93 (0.50, 17.11)	2	28	3	0	0.23
≤5.5 versus >5.5	2.64 (0.59, 11.83)	3	120	19	0	0.21
≤6.0 versus >6.0	2.93 (0.50, 4.25)	2	28	3	0	0.76

NA, not applicable.

**Table 7.** Summary of subgroup analysis for hepatotoxicity

Subgroup		Cut-off value (mg/L)	RR (95% CI)	No. of studies	No. of participants in experimental group	No. of participants in control group	*I*^2^ %	*P*
Study location	Asian location	≤3.0 versus >3.0	0.31 (0.16, 0.63)	4	142	80	20	0.001
		≤4.0 versus >4.0	0.27 (0.11, 0.63)	5	213	77	66	0.003
		≤5.0 versus >5.0	0.34 (0.13, 0.87)	4	190	32	72	0.02
		≤5.5 versus >5.5	0.36 (0.17, 0.74)	4	193	29	51	0.006
		≤6.0 versus >6.0	0.36 (0.21, 0.63)	5	217	30	24	<0.001
	non-Asian location	≤3.0 versus >3.0	2.50 (0.27, 22.86)	1	8	10	NA	0.42
		≤4.0 versus >4.0	3.77 (0.23, 63.05)	1	12	6	NA	0.36
		≤5.0 versus >5.0	3.00 (0.18, 49.56)	1	13	5	NA	0.44
		≤5.5 versus >5.5	0.55 (0.27, 1.15)	4	203	62	0	0.11
		≤6.0 versus >6.0	0.48 (0.22, 1.06)	2	119	21	0	0.07
Population	children	≤3.0 versus >3.0	0.47 (0.01, 16.92)	2	21	18	75	0.68
		≤4.0 versus >4.0	0.62 (0.02, 17.57)	2	27	12	73	0.78
		≤5.0 versus >5.0	0.59 (0.02, 14.84)	2	31	8	76	0.75
		≤5.5 versus >5.5	0.59 (0.02, 14.84)	2	31	8	76	0.75
		≤6.0 versus >6.0	0.26 (0.06, 1.03)	2	35	4	7	0.05
	adults	≤3.0 versus >3.0	0.35 (0.18, 0.68)	3	129	72	16	0.002
		≤4.0 versus >4.0	0.29 (0.11, 0.73)	5	198	71	71	0.009
		≤5.0 versus >5.0	0.40 (0.13, 1.18)	3	172	29	78	0.10
		≤5.5 versus >5.5	0.47 (0.31, 0.72)	6	365	83	0	<0.001
		≤6.0 versus >6.0	0.44 (0.29, 0.66)	5	301	47	0	<0.001

NA, not applicable.

**Figure 5.**
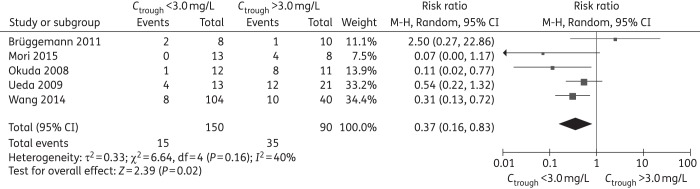
Meta-analysis for incidence of hepatotoxicity (trough concentration of <3.0 mg/L comparison with >3.0 mg/L, RR <1 favours *C*_trough_ <3.0 mg/L).

**Figure 6.**
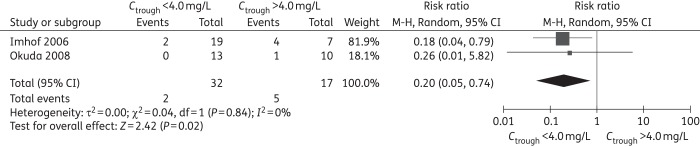
Meta-analysis for incidence of neurotoxicity (trough concentration of <4.0 mg/L comparison with >4.0 mg/L, RR <1 favours *C*_trough_ <4.0 mg/L).

For neurotoxicity, the meta-analysis demonstrated a significant increase at voriconazole trough cut-off values >4.0 mg/L (RR = 0.20, 95% CI 0.05–0.74) and >5.5 mg/L (RR = 0.37, 95% CI 0.21–0.65) (Table 6 and Figure 6). Owing to the scant data, subgroup analysis was performed only at a cut-off level of 5.5 mg/L, which showed the incidence of neurotoxicity was significantly increased in the non-Asian study locations (RR = 0.36, 95% CI 0.17–0.75).

For visual disorders, there were no significant differences in incidence between the interventional and control groups at all cut-off levels (Table 6).

Sensitivity analysis on each study's effect on the summary estimates showed that exclusion of studies by Wang *et al*.,^12^ Okuda *et al*.^42^ or Ueda *et al*.,^14^ resulted in an insignificant difference at a cut-off level of 3.0 mg/L. Exclusion of studies by Ueda *et al*.^14^ or Brüggemann *et al*.^35^ resulted in a significant increased incidence of hepatotoxicity at trough concentration >5.0 mg/L. Additionally, exclusion of the study by Koselke *et al*.^30^ resulted in a significant increased incidence of neurotoxicity at trough concentration >5.5 mg/L (Table S11).

### Publication bias and sensitivity analysis

Owing to the limited number of studies, we only evaluated publication bias at the trough concentration cut-off level of 1 mg/L for treatment success (10 studies). The results of Begg's test (*P* = 0.929) and Egger's test (*P* = 0.539) showed a low likelihood of publication bias.

### Assessment of quality of included studies

Using the nine-point scoring system, most studies scored between 7 and 8. Assessment of study-specific quality scores from the Newcastle–Ottawa Scale system is summarized in Table S12.

## Discussion

### Efficacy

Major guidelines support and recommend TDM for voriconazole,^2–6^ although exact threshold levels remain inconclusive. We determined in this meta-analysis that a trough concentration of 0.5 mg/L is associated with efficacy, which differs from the 1.0–2.0 mg/L threshold recommended in some publications.^16,45^ Our findings are similar to the results in an FDA analysis of 280 patients, which suggested a trend to higher success rates in patients with mean voriconazole levels >0.5 mg/L.^46^ Our subgroup analysis for patients with proven or probable IFI, patients on monotherapy and a sensitivity analysis based on individual patient data further validated the 0.5 mg/L trough concentration for efficacy (Table 4). First, ‘possible IFI’ is less reliable than a proven or probable IFI diagnosis and has limited value in clinical trials because it does not require mycological evidence, and host factors and clinical features are not sufficiently specific, resulting in the inclusion of non-IFI patients.^47^ Besides, five studies included persistently febrile neutropenic patients^10,14,31,35,42^ and used voriconazole as empirical therapy. This may explain why voriconazole did not show significant improvement in treatment success rates. Secondly, antifungal combination therapy with micafungin, caspofungin or amphotericin B may confound the assessment of an exposure–response relationship of voriconazole,^48^ which might explain why the combo therapy subgroup did not show significance at the 0.5 mg/L cut-off level.

This 0.5 mg/L cut-off value is in conflict with the results of a study in 825 patients,^22^ which suggested a trough/MIC (MIC_90_ for the majority of fungal pathogens is 0.5–1 mg/L^26^) ratio of 2–5 as a TDM target based on Monte Carlo simulation. Notably, in clinical practice, fungal voriconazole MIC data are usually not available, which limits the utility of this metric.^48^ Furthermore, the conclusion of this study was based on simulated data rather than real patients' data. Thus, we believe our result has greater validity.

Sensitivity analysis showed that the rate of treatment success significantly decreased at a cut-off level of <1.5 mg/L when excluding the study by Kim *et al*.,^29^ hence future studies are needed to test our conclusion of the 0.5 mg/L lower limit further.

A number of factors contribute to the death of patients with IFI, such as progress of underlying disease, concomitant infection and severe adverse effects. Although our result showed significantly decreased all-cause mortality at a cut-off level of <3.0 mg/L, the sample size was small (only two studies contributed data) and the confounding factors could not be removed. Thus, the significance of our result for all-cause mortality is likely unreliable.

### Safety

It seems reasonable that the most severe adverse event, hepatotoxicity, should be the focus since other events occur less frequently or have limited lasting sequelae. Our meta-analysis indicated a concentration of >3.0 mg/L is associated with an increased risk of hepatotoxicity, which is considerably lower than described in previous studies.^16,18^ Subgroup analysis found the incidence of hepatotoxicity in the studies conducted in Asia were different than non-Asian studies, suggesting the possibility that the concentration–hepatotoxicity relationship follows a different profile among different races. Sensitivity analysis showed the incidence of hepatotoxicity became insignificant at a cut-off of 3.0 mg/L when removing the studies by Okuda *et al*.,^42^ Wang *et al*.^12^ or Ueda *et al*.^14^ Notably, these three studies were all conducted in a predominantly Asian population. Therefore, a lowered upper limit of the target concentration should be considered for Asian patients compared with the upper limit for non-Asian patients. Voriconazole does exhibit high inter- and intra-patient variability in the pharmacokinetic profile following oral and intravenous doses.^21,49^ Because of the variability, a reasonable recommendation for treatment would be to obtain a trough concentration once steady state is achieved, with target concentrations between 0.5 and 3.0 mg/L. Clearly, adequately powered, prospective, multicentre research is needed to answer these important questions.

### Strengths and limitations

Our study has several strengths. First, this meta-analysis allowed comparison of commonly used cut-off levels for efficacy and safety in a single analysis for individual cut-off levels. Second, we used explicit, pre-defined efficacy and safety outcomes to minimize heterogeneity of outcomes across different studies. Finally, we obtained additional and individual data from the study authors to perform more detailed analyses (e.g. extracting individual data for patients on monotherapy).

We acknowledge the following limitations to our work. First, due to the paucity of available data, a detailed analysis according to pathological condition (e.g. whether resistant to voriconazole or not) or infection location was not performed. In addition, we were unable to perform subgroup analysis for different patient populations and some results remain inconclusive. Besides, rare, serious adverse events such as renal failure and cardiotoxicity were not evaluated. Second, the use of observational studies in a meta-analysis is prone to biases and confounding factors that are inherent in the original studies. Third, differences in assay methods across studies may lead to differences in precision of the voriconazole result and differences in the timing of clinical outcome assessment may lead to lack of reliability in the results across studies.

### Conclusions

This meta-analysis demonstrated that 0.5 mg/L is the lower limit of the target voriconazole trough concentration during treatment. Trough concentrations of >3.0 mg/L are associated with an increased risk of moderate–severe hepatotoxicity, particularly for the Asian population. Trough concentrations >4.0 mg/L were associated with an increased risk of neurotoxicity.

## Funding

This study was carried out as part of our routine work.

## Transparency declarations

B. A. F. was a co-investigator on a project for which the principal investigator received research support from HOSPIRA. All other authors: none to declare.

### Author contributions

H. J. performed searches, screened search results, screened retrieved papers against inclusion criteria, extracted data from papers, wrote to authors of papers for additional information, provided additional data from papers, managed data for the review, did analysis and interpretation of data and drafted the manuscript. T. W. designed and coordinated the review, did interpretation of data, prepared the manuscript and provided general advice on the review. B. A. F. provided a clinical perspective, contributed to data interpretation and revised the manuscript. K. M. O. provided a clinical perspective, contributed to data interpretation and revised the manuscript. K. C. conducted searching, extracted data and appraised the quality of papers. H. T. provided a methodological perspective on meta-analysis. J. H. provided a clinical perspective and revised the manuscript. S. Z. conceived and coordinated the review. All authors read and approved the final version of the manuscript.

## Supplementary data

Tables S1–S12 and Figures S1–S55 are available as Supplementary data at *JAC* Online (http://jac.oxfordjournals.org/).

Supplementary Data
