# Supplementary material for: Trough concentration of voriconazole and its relationship with efficacy and safety: a systematic review and meta-analysis
Source: J Antimicrob Chemother. 2016 Mar 10;71(7):1772–85. doi: 10.1093/jac/dkw045 (PMC4896404; doi:10.1093/jac/dkw045)
Supplement: Supplementary Data [file supp_71_7_1772__index.html]

Trough concentration of voriconazole and its relationship with efficacy and safety: a systematic review and meta-analysis — Trough concentration of voriconazole and its relationship with efficacy and safety: a systematic review and meta-analysis — Supplementary Data 

# Trough concentration of voriconazole and its relationship with efficacy and safety: a systematic review and meta-analysis

## Supplementary Data

Supplementary Data

- Supplementary Data - Doc file
