# Supplementary material for: Trough concentration of voriconazole and its relationship with efficacy and safety: a systematic review and meta-analysis
Source: J Antimicrob Chemother. 2016 Mar 10;71(7):1772–85. doi: 10.1093/jac/dkw045 (PMC4896404; doi:10.1093/jac/dkw045)
Supplement: Supplementary Data [file supp_dkw045_dkw045supp.doc]

**Supplementary data**

**Contents**

**Appendix 1 Definitions for outcomes (Table S1)**

**Appendix 2 The reason for excluding observational studies (Table S2)**

**Appendix 3 Raw data for efficacy (Table S3-S6)**

**Appendix 4 Sensitivity analysis on each study’s affect on the summary estimates for efficacy (Table S7)**

**Appendix 5 Raw data for safety (Table S8-S10)**

**Appendix 6 Sensitivity analysis on each study’s affect on the summary estimates for safety (Table S11)**

**Appendix 7 Quality appraisal of included studies (Table S12)**

**Appendix 8 Forest plot for efficacy and safety at each cutoff value (Figure S1-S55)**

**8.1 Forest plot for rate of treatment success at each cutoff value (Figure S1-S4)**

**8.2 Forest plot for subgroup analysis of rate of treatment success at each cutoff value (Figure S5-S19)**

**8.3 Forest plot for sensitivety analysis of treatment success at each cutoff value (Figure S20-S23)**

**8.4 Forest plot for all cause mortality at each cutoff value (Figure S24-S27)**

**8.5 Forest plot for incidence of IFI at each cutoff value (Figure S28-S32)**

**8.6 Forest plot for safety at each cutoff value (Figure S33-S55)**

**Appendix 1 Definitions for outcomes (Table S1)**

| **Outcomes** | **Defections** |
| --- | --- |
| Invasive fungal infection (IFI) | The IFI was defined according to the European Organization for Research and Treatment of Cancer/Invasive Fungal Infections Cooperative Group and the National Institute of Allergy and Infectious Diseases Mycoses Study Group (EORTC/MSG) criteria[^28^](#_ENREF_28). |
| Treatment success* (Complete and partial response) | Defined as partial resolution or resolution of clinically significant signs and symptoms associated with fungal infection, improvement or resolution of computed tomography (CT) or magnetic resonance imaging (MRI) findings, and on proven or negative culture results. |
| Hepatotoxicity | Defined as when the grade (for the reported scale) of a liver function test (like Aspartate transaminase (AST), Alanine aminotransferase(ALT,) alkaline phosphatase (ALP),γ-glutamyl transpeptidase(γ-GTP), or Bilirubin) after VRC initiation increased compared to the grade determined before VRC initiation. |
| Neurotoxicity | Included encephalopathy, auditory hallucinations, visual hallucinations, confusion and seizures. |
| Visual disorder | Defined as photophobia, blurred vision, color blindness, optic neuritis, or papilledema. |

***** Given the known variation in the definitions of treatment success in the literature, we used the criteria from the majority of included studies to minimize heterogeneity (complete and partial response). Definition of treatment success in each included study was shown in Table 2 in the manuscript.

**Appendix 2 The reason for excluding observational studies (Table S2)**

| **No** | **Excluded studies** | **Reason for exclusion** |
| --- | --- | --- |
| 1 | Markantonis SL, Katelari A, Pappa E, Doudounakis S. Voriconazole pharmacokinetics and photosensitivity in children with cystic fibrosis. J Cyst Fibros. 2012. 11(3): 246-52. | Data is from pharmacokinetics model rather than from real patients |
| 2 | Berge M, Guillemain R, Boussaud V, et al. Voriconazole pharmacokinetic variability in cystic fibrosis lung transplant patients. Transpl Infect Dis. 2009. 11(3): 211-9. | A lack of outcome data at defined cutoff levels. |
| 3 | Barreto JN, Beach CL, Wolf RC, et al. The incidence of invasive fungal infections in neutropenic patients with acute leukemia and myelodysplastic syndromes receiving primary antifungal prophylaxis with voriconazole. Am J Hematol. 2013. 88(4): 283-8. | A lack of cutoff value of trough concentration |
| 4 | Park, Kim N, Kim K, Lee SH, et al.The effect of therapeutic drug monitoring on safety and efficacy of voriconazole in invasive fungal infections: A randomized controlled trial. Clinical Infectious Diseases. 2012. 55(8): 1080-1087 | A lack of outcome data in corresponding concentration |
| 5 | Hoenigl M, Duettmann W, Raggam RB, et al. Potential factors for inadequate voriconazole plasma concentrations in intensive care unit patients and patients with hematological malignancies. Antimicrob Agents Chemother. 2013. 57(7): 3262-7 | A lack of cutoff value of trough concentration |
| 6 | Eiden C, Cociglio M, Hillaire-Buys D, et al. Pharmacokinetic variability of voriconazole and N-oxide voriconazole measured as therapeutic drug monitoring. Xenobiotica. 2010. 40(10): 701-6. | A lack of data to estimate the rate of efficacy and safety at both below and above the cutoff value |
| 7 | Dolton MJ, Ray JE, Chen SC-A, Ng K, Pont LG. Multicenter study of voriconazole pharmacokinetics and therapeutic drug monitoring. Antimicrob Agents Chemother. 2012. 56(9): 4793-9 | Data is from ROC analysis rather than from real patients |
| 8 | Matsumoto K, Ikawa K, Abematsu K, et al. Correlation between voriconazole trough plasma concentration and hepatotoxicity in patients with different CYP2C19 genotypes. Int J Antimicrob Agents. 2009. 34(1): 91-4. | Data is from pharmacokinetics model rather than from real patients |
| 9 | Lombardi LR, Miano TA, Davis JL, et al. A retrospective analysis of the effect of patient-specific factors on voriconazole concentrations in oncology patients. J Oncol Pharm Pract. 2012. 18(1): 3-9. | A lack of outcome data in corresponding concentration |
| 10 | Tan K, Brayshaw N, Tomaszewski K, Troke P, Wood N. Investigation of the potential relationships between plasma voriconazole concentrations and visual adverse events or liver function test abnormalities. J Clin Pharmacol. 2006. 46(2): 235-43 | Data is from pharmacokinetics model rather than from real patients |
| 11 | Narita A, Muramatsu H, Sakaguchi H, et al. Correlation of CYP2C19 phenotype with voriconazole plasma concentration in children. J Pediatr Hematol Oncol. 2013. 35(5): e219-23 | A lack of data to estimate the rate of efficacy and safety at both below and above the cutoff value |
| 12 | Bartelink IH, Wolfs T, Jonker M, et al. Highly variable plasma concentrations of voriconazole in pediatric hematopoietic stem cell transplantation patients. Antimicrob Agents Chemother. 2013. 57(1): 235-40. | A lack of outcome data at defined cutoff levels. |
| 13 | Choi SH, Lee SY, Hwang JY, et al. Importance of voriconazole therapeutic drug monitoring in pediatric cancer patients with invasive aspergillosis. Pediatr Blood Cancer. 2013. 60(1): 82-7 | The number of below and above cufoff value were estimated by samples rather than included patients |
| 14 | Gerin M, Mahlaoui N, Elie C, et al. Therapeutic drug monitoring of voriconazole after intravenous administration in infants and children with primary immunodeficiency. Ther Drug Monit. 2011. 33(4): 464-6 | A lack of outcome data at defined cutoff levels. |
| 15 | Soler-Palacin P, Frick MA, Martin-Nalda A, et al. Voriconazole drug monitoring in the management of invasive fungal infection in immunocompromised children: a prospective study. J Antimicrob Chemother. 2012. 67(3): 700-6 | The number of below and above cufoff value were estimated by samples rather than the included patients |
| 16 | Hicks JK. Voriconazole plasma concentrations in immunocompromised pediatric patients vary by CYP2C19 diplotypes. PharmacogenomicsPharmacogenomics. 2014. 15(8): 1065 | A lack of outcome data |
| 17 | Mori M, Fukushima K, Miharu M, Goto H, Yoshida M, Shoji S. A retrospective analysis of voriconazole pharmacokinetics in Japanese pediatric and adolescent patients. J Infect Chemother. 2013. 19(1): 174-9 | The number of below and above cufoff value were estimated by a sample rather than the included patients |
| 18 | Baxter CG, Marshall A, Roberts M, Felton TW, Denning DW. Peripheral neuropathy in patients on long-term triazole antifungal therapy. J Antimicrob Chemother. 2011. 66(9): 2136-9. | Small sample size. |
| 19 | Davies-Vorbrodt S, Ito JI, Tegtmeier BR, Dadwal SS, Kriengkauykiat J. Voriconazole serum concentrations in obese and overweight immunocompromised patients: a retrospective review. Pharmacotherapy. 2013. 33(1): 22-30. | Small sample size. |
| 20 | [Saini L](http://www.ncbi.nlm.nih.gov/pubmed/?term=Saini L%5BAuthor%5D&cauthor=true&cauthor_uid=25371690), [Seki JT](http://www.ncbi.nlm.nih.gov/pubmed/?term=Seki JT%5BAuthor%5D&cauthor=true&cauthor_uid=25371690), [Kumar D](http://www.ncbi.nlm.nih.gov/pubmed/?term=Kumar D%5BAuthor%5D&cauthor=true&cauthor_uid=25371690), et al. Serum voriconazole level variability in patients with hematological malignancies receiving voriconazole therapy. [Can J Infect Dis Med Microbiol.](http://www.ncbi.nlm.nih.gov/pubmed/?term=Serum+voriconazole+level+variability+in+patients+with+hematological+malignancies+receiving+voriconazole+therapy) 2014,25(5):271-6. | A lack of data to estimate the rate of efficacy and safety at both below and above the cutoff value |
| 21 | Troke PF, Hockey HP, Hope WW. Observational study of the clinical efficacy of voriconazole and its relationship to plasma concentrations in patients. Antimicrob Agents Chemother. 2011. 55(10): 4782-8 | Concentration reported was not trough concentration |
| 22 | Smith J, Safdar N, Knasinski V, et al. Voriconazole therapeutic drug monitoring. Antimicrob Agents Chemother. 2006. 50(4): 1570-2 | Concentration reported was random concentration |
| 23 | Zonios D, Yamazaki H, Murayama N, et al. Voriconazole metabolism, toxicity, and the effect of cytochrome P450 2C19 genotype. J Infect Dis. 2014. 209(12): 1941-8 | A lack of cutoff value of trough concentration. |
| 24 | Lutsar I, Roffey S, Troke P. Voriconazole concentrations in the cerebrospinal fluid and brain tissue of guinea pigs and immunocompromised patients. Clin Infect Dis. 2003. 37(5): 728-32 | Concentration reported was not trough concentration or at steady state. |
| 25 | Miyakis S, van Hal SJ, Solvag CJ, Ray J, Marriott D. Clinician ordering practices for voriconazole therapeutic drug monitoring: experiences of a referral laboratory. Ther Drug Monit. 2010. 32(5): 661-4 | Concentration reported was not trough concentration |
| 26 | Neely M, Rushing T, Kovacs A, Jelliffe R, Hoffman J. Voriconazole pharmacokinetics and pharmacodynamics in children. Clin Infect Dis. 2010. 50(1): 27-36. | The tough concentrations could not be extracted and it is unclear that weather the concentrations were at steady state. |
| 27 | Doby EH, Benjamin DK Jr, Blaschke AJ, et al. Therapeutic monitoring of voriconazole in children less than three years of age: a case report and summary of voriconazole concentrations for ten children. Pediatr Infect Dis J. 2012. 31(6): 632-5. | Inappropriate study design (case report) |
| 28 | Miyakis S, van Hal SJ, Ray J et al. Voriconazole concentrations and outcome of invasive fungal infections. Clin Microbiol Infect 2010;16:927-33. | Cut off value reported were 0.35mg/L and 2.2mg/L, did not meet our pre-defined criteria |

**Appendix 3 Raw data for efficacy (Table S3-S6)**

**Table S3 Raw data for rate of treatment success with different cutoff value**

| **Reference** | **0.5 mg/l** | | **1mg/l** | | **1.5mg/l** | | **2mg/l** | | **3mg/l** | |
| --- | --- | --- | --- | --- | --- | --- | --- | --- | --- | --- |
|  | **≤0.5** | **＞0.5** | **≤1** | **＞1** | **≤1.5** | **＞1.5** | **≤2.0** | **＞2.0** | **≤3.0** | **＞3.0** |
| Brüggemann 2011 | NR | NR | 1/2 | 7/11 | 3/4 | 5/9 | 5/6 | 3/7 | 6/8 | 2/5 |
| ^※^Brüggemann 2011 | NR | NR | 1/2 | 6/8 | 3/4 | 4/6 | 5/6 | 2/4 | 6/7 | 1/3 |
| Denning 2002 | 3/11 | 58/111 | NR | NR | NR | NR | NR | NR | NR | NR |
| Kim 2013 | 1/2 | 71/102 | 9/11 | 63/93 | 21/25 | 51/79 | 30/37 | 42/67 | 55/72 | 17/32 |
| ^a^Lee 2013 | 2/5 | 24/47 | 2/6 | 24/46 | NR | NR | 5/11 | 21/41 | 10/17 | 16/35 |
| ^b^Lee 2013 | 2/5 | 29/47 | 2/6 | 29/46 | NR | NR | 5/11 | 26/41 | 11/17 | 20/35 |
| ^c^Lee 2013 | 2/5 | 28/47 | 2/6 | 28/46 | NR | NR | 5/11 | 25/41 | 9/17 | 21/35 |
| ^d^Lee 2013 | 2/5 | 25/47 | 2/6 | 25/46 | NR | NR | 5/11 | 22/41 | 7/17 | 20/35 |
| ^e^Chu 2013 | NR | NR | 7/13 | 6/33 | NR | NR | NR | NR | NR | NR |
| ^d^Chu 2013 | NR | NR | 9/13 | 13/33 | NR | NR | NR | NR | NR | NR |
| Okuda 2008 | 0/3 | 4/18 | 0/5 | 4/16 | 0/7 | 4/14 | 0/9 | 4/12 | 0/10 | 4/11 |
| ^※^Okuda 2008 | 0/1 | 4/13 | 0/3 | 4/11 | 0/4 | 4/10 | 0/5 | 4/9 | 0/6 | 4/8 |
| Psacual 2008 | NR | NR | 7/13 | 34/39 | NR | NR | NR | NR | NR | NR |
| Racil 2012 | NR | NR | 15/17 | 22/36 | NR | NR | 27/36 | 10/17 | NR | NR |
| Wang 2014 | 4/15 | 114/129 | 24/42 | 94/102 | 48/68 | 70/76 | 63/84 | 55/60 | 81/104 | 37/40 |
| Ueda 2009 | 0/1 | 23/33 | 1/3 | 22/31 | 4/7 | 19/27 | 7/10 | 16/24 | 13/17 | 10/17 |
| Gomez 2012 | 1/4 | 8/10 | 3/7 | 6/7 | 5/9 | 4/5 | 6/11 | 3/3 | 8/13 | 1/1 |
| ^※^Gomez 2012 | 1/2 | 4/4 | 2/3 | 3/3 | 3/4 | 2/2 | 3/4 | 2/2 | 4/5 | 1/1 |
| ^＃^Gomez 2012 | 1/2 | 6/8 | 2/4 | 5/6 | 4/6 | 3/4 | 5/8 | 2/2 | 6/9 | 1/1 |

NR=not reported.

^a^ 2-week response.

^b^4-week response.

^c^8-week response.

^d^12-week response.

^e^6-week response

^※^excluded patients on concomitant use of other antifungals.

^＃^excluded children.

**Table S4 Raw data for IFI related mortality with different cutoff values.**

| **Reference** | **0.35 mg/l** | | | **0.5 mg/l** | | | | **1mg/l** | | **1.5mg/l** | | | **2mg/l** | | | **3mg/l** | | |
| --- | --- | --- | --- | --- | --- | --- | --- | --- | --- | --- | --- | --- | --- | --- | --- | --- | --- | --- |
|  | **≤0.35** | **＞0.35** | | **≤0.5** | **＞0.5** | | | **≤1** | **＞1** | **≤1.5** | **＞1.5** | | **≤2.0** | | **＞2.0** | **≤3.0** | **＞3.0** | |
| VRC used for treatment | | | | | | | | | | | | | | | | | | |
| Kim 2013 | NR | NR | | 0/2 | | 10/102 | | 0/11 | 10/93 | 0/25 | 10/79 | | 1/37 | | 9/67 | 5/72 | | 5/32 |
| VRC used for prevent from fungal disease | | | | | | | | | | | | | | | | | | |
| trifilio 2007 | 0/15 | | 4/56 | 1/28 | | | 3/43 | NR | NR | NR | | NR | | 3/41 | 1/30 | NR | | NR |

VRC = voriconazole; NR=not reported

**Table S5 Raw data for all cause** mortality with different cutoff value

| **Reference** | **0.35 mg/l** | | | **0.5 mg/l** | | | | **1mg/l** | | **1.5mg/l** | | **2mg/l** | | | | | **3mg/l** | | | |
| --- | --- | --- | --- | --- | --- | --- | --- | --- | --- | --- | --- | --- | --- | --- | --- | --- | --- | --- | --- | --- |
|  | **≤0.35** | **＞0.35** | | **≤0.5** | **＞0.5** | | | **≤1** | **＞1** | **≤1.5** | **＞1.5** | **≤2.0** | | **＞2.0** | | | **≤3.0** | | **＞3.0** | |
| VRC used for treatment | | | | | | | | | | | | | | | | | | | | |
| Kim 2013 | 0/1 | 21/103 | | 0/2 | | 21/102 | | 1/11 | 20/93 | 2/25 | 19/79 | 4/37 | | 17/67 | | | 10/72 | | | 11/32 |
| Gomez 2012 | 2/3 | 2/11 | | 3/4 | | 1/10 | | 3/7 | 1/7 | 3/9 | 1/5 | 4/11 | | 0/3 | | | 4/13 | | | 0/1 |
| VRC used for prevent from fungal disease | | | | | | | | | | | | | | | | | | | | |
| trifilio 2007 | 0/15 | | 4/56 | 1/28 | | | 3/43 | NR | NR | NR | NR | | 3/41 | | 1/30 | NR | | NR | | |

VRC = voriconazole; NR=not reported

**Table S6 Raw data for incidence of IFI** **with different cutoff values.**

| **Reference** | **0.5 mg/l** | | **1mg/l** | | **1.5mg/l** | | **2mg/l** | | **3mg/l** | | **5mg/l** | |
| --- | --- | --- | --- | --- | --- | --- | --- | --- | --- | --- | --- | --- |
|  | **≤0.5** | **＞0.5** | **≤1** | **＞1** | **≤3.0** | **＞3.0** | **≤2.0** | **＞2.0** | **≤3.0** | **＞3.0** | **≤5.0** | **＞5.0** |
| Mitsani D 2012 | NR | NR | 5/33 | 5/60 | 6/37 | 4/56 | NR | NR | NR | NR | NR | NR |
| Trifilio 2007 | 3/15 | 7/56 | 5/28 | 5/43 | NR | NR | 7/41 | 3/30 | NR | NR | 9/65 | 1/8 |
| Heng 2013 | 2/4 | 2/8 | 2/7 | 2/5 | 2/7 | 2/5 | 2/9 | 2/3 | 2/9 | 2/3 | 4/12 | 0 |
| Brüggemann 2010 | 0/1 | 1/9 | 1/4 | 0/6 | 1/6 | 0/4 | 1/7 | 0/3 | 1/9 | 0/1 | 1/9 | 0/1 |

NR=not reported

**Appendix 4 Sensitivity analysis on each study’s affect on the summary estimates for efficacy (Table S7)**

**Table S7 Sensitivity analysis on each study’s affect on the summary estimates for efficacy (results were only reported when differing from primary analysis)**

| **Cutoff value** | **Rate of treatment success** | | | **All cause mortality** | | | **Incidence of IFI** | | |
| --- | --- | --- | --- | --- | --- | --- | --- | --- | --- |
|  | **Sensitivity analysis** | **No. of studies attributing data for sensitivity analysis** | **Primary**  **analysis** | **Sensitivity analysis** | **No. of studies attributing data for sensitivity analysis** | **Primary**  **analysis** | **Sensitivity analysis** | **No. of studies attributing data for sensitivity analysis** | **Primary**  **analysis** |
| 0.5 | - | 6 | 0.46 [0.29, 0.74], I^2^%=0 | - | 1 | 2.87 [0.32, 25.52], I^2^%=47 | - | 2 | 1.74 [0.70, 4.31], I^2^%=0 |
| 1.0 | - | 9 | 0.88 [0.61, 1.26], I^2^%=73 | - | 1 | 1.10 [0.16, 7.68], I^2^%=49 | - | 3 | 1.49 [0.73, 3.01], I^2^%=0 |
| 1.5 | 0.78 [0.67, 0.91], I^2^%=0 ^**^ | 5 | 0.93 [0.67, 1.30], I^2^%=68 | - | 1 | 0.64 [0.13, 3.06], I^2^%=43 | - | 2 | 1.55 [0.62, 3.84], I^2^%=0 |
| 2.0 | - | 7 | 1.01 [0.78, 1.30], I^2^%=62 | - | 1 | 0.75[0.13, 4.27], I^2^%=44 | - | 2 | 0.88 [0.26, 2.95], I^2^%=35 |
| 3.0 | - | 6 | 1.03 [0.75, 1.42], I^2^%=62 | - | 1 | 0.44[0.22, 0.91], I^2^%=0 | - | 1 | 0.38 [0.10, 1.38], I^2^%=0 |

IFI=invasive fungal infection; -,=no meaningful difference

**,after removing study by Kim

**Appendix 5 Raw data for safety (Table S8-S10)**

**Table S8 Raw data for incidence of hepatotoxicity with different cutoff values.**

| **Reference** | **3.0mg/l** | | **4.0mg/l** | | **5.0mg/l** | | **5.5mg/l** | | **6mg/l** | |
| --- | --- | --- | --- | --- | --- | --- | --- | --- | --- | --- |
|  | **≤3.0** | **＞3.0** | **≤4.0** | **＞4.0** | **≤5.0** | **＞5.0** | **≤5.5** | **＞5.5** | **≤6.0** | **＞6.0** |
| Brüggemann 2011 | 2/8 | 1/10 | 3/12 | 0/6 | 3/13 | 0/5 | 3/13 | 0/5 | 3/17 | 0/1 |
| Chu 2013 | NR | NR | NR | NR | NR | NR | 11/92 | 4/16 | NR | NR |
| Denning 2002 | NR | NR | NR | NR | NR | NR | NR | NR | 14/100 | 6/22 |
| Kim 2011 | NR | NR | NR | NR | NR | NR | NR | NR | 3/20 | 2/5 |
| Koselke 2012 | NR | NR | NR | NR | NR | NR | 3/62 | 2/25 | NR | NR |
| Okuda 2008 | 1/12 | 8/11 | 1/13 | 8/10 | 3/15 | 6/8 | 3/15 | 6/8 | 3/15 | 6/8 |
| Psacual 2008 | NR | NR | NR | NR | NR | NR | 3/36 | 3/16 | NR | NR |
| Suzuki 2013 | NR | NR | 4/25 | 7/14 | NR | NR | NR | NR | NR | NR |
| Wang 2014 | 8/104 | 10/40 | 11/124 | 7/20 | 14/136 | 4/8 | 15/137 | 3/7 | 16/140 | 2/4 |
| Qu 2013 | 1/4 | 2/6 | 1/4 | 2/6 | 1/5 | 2/5 | 1/6 | 2/4 | 1/6 | 2/4 |
| Mori 2015 | 0/13 | 4/8 | 1/15 | 3/6 | 2/18 | 2/3 | 2/18 | 2/3 | 2/18 | 2/3 |
| Ueda 2009 | 4/13 | 12/21 | 9/19 | 7/15 | 10/21 | 6/13 | 10/23 | 6/11 | 10/24 | 6/10 |
| Matsumoto 2009 | NR | NR | 1/17 | 9/12 | NR | NR | NR | NR | NR | NR |

NR=not reported

**Table S9 Raw data for incidence of neurotoxicity with different cutoff values.**

| **Reference** | **3.0mg/l** | | **4.0mg/l** | | **5.0mg/l** | | **5.5mg/l** | | **6mg/l** | |
| --- | --- | --- | --- | --- | --- | --- | --- | --- | --- | --- |
|  | **≤3.0** | **＞3.0** | **≤4.0** | **＞4.0** | **≤5.0** | **＞5.0** | **≤5.5** | **＞5.5** | **≤6.0** | **＞6.0** |
| Chu 2013 | NR | NR | NR | NR | NR | NR | 15/92 | 6/16 | NR | NR |
| Kim 2011 | NR | NR | NR | NR | NR | NR | NR | NR | 1/20 | 0/5 |
| Koselke 2012 | NR | NR | NR | NR | NR | NR | 9/80 | 8/28 | NR | NR |
| Okuda 2008 | 0/12 | 1/11 | 0/13 | 1/10 | 0/15 | 1/8 | 0/15 | 1/8 | 0/15 | 1/8 |
| Psacual 2008 | NR | NR | NR | NR | NR | NR | 0/36 | 5/16 | NR | NR |
| Fu 2013 | 0/8 | 1/11 | 0/10 | 1/9 | 1/14 | 0/5 | 1/14 | 0/5 | 1/14 | 0/5 |
| Imhof 2006 | 2/12 | 4/14 | 2/19 | 4/7 | NR | NR | NR | NR | NR | NR |

NR=not reported

**Table S10 Raw data for incidence of visual disturbance with different cutoff values**

| **Reference** | **3.0mg/l** | | **4.0mg/l** | | **5.0mg/l** | | **5.5mg/l** | | **6mg/l** | |
| --- | --- | --- | --- | --- | --- | --- | --- | --- | --- | --- |
|  | **≤3.0** | **＞3.0** | **≤4.0** | **＞4.0** | **≤5.0** | **＞5.0** | **≤5.5** | **＞5.5** | **≤6.0** | **＞6.0** |
| Chu 2013 | NR | NR | NR | NR | NR | NR | 5/92 | 0/16 | NR | NR |
| Mori 2015 | 7/15 | 2/6 | 9/17 | 0/4 | 9/19 | 0/2 | 9/19 | 0/2 | 9/19 | 0/2 |
| Brüggemann 2010 | 7/9 | 0/1 | 7/9 | 0/1 | 7/9 | 0/1 | 7/9 | 0/1 | 7/9 | 0/1 |

NR=not reporte

**Appendix 6 Sensitivity analysis on each study’s affect on the summary estimates for safety (Table S11)**

**Table S11 Sensitivity analysis on each study’s affect on the summary estimates for safety (results only reported when the conclusions differed from primary analysis)**

| **Cutoff value** | **Hepatotoxicity** | | | **Neurotoxicity** | | | **Visual disturbance** | | |
| --- | --- | --- | --- | --- | --- | --- | --- | --- | --- |
|  | **Sensitivity analysis** | **No. of studies attributing data for sensitivity analysis** | **Primary**  **analysis** | **Sensitivity analysis** | **No. of studies attributing data for sensitivity analysis** | **Primary**  **analysis** | **Sensitivity analysis** | **No. of studies attributing data for sensitivity analysis** | **Primary**  **analysis** |
| 3.0 | 1. 10.44 [0.19, 1.03], I^2^%=38^a^  2. 0.37 [0.10, 1.41], I^2^%=53^b^  3.0.30 [0.09, 1.01]  I^2^%=46^c^ | 4 | 0.37 [0.16, 0.83], I^2^%=40 | - | 1 | 0.52 [0.13, 2.01], I^2^%=0 | - | 1 | 1.64 [0.54, 5.01], I^2^%=0 |
| 4.0 | - | 6 | 0.32 [0.14, 0.74], I^2^%=64 | 0.26 [0.01, 5.82], I^2^%=NA^d^ | 1 | 0.20 [0.05, 0.74], I^2^%=0 | - | 1 | 3.88 [0.64, 23.32], I^2^%=0 |
| 5.0 | 1. 0.26 [0.12, 0.57], I^2^%=31^c^  2. 0.34 [0.13, 0.87], I^2^%=72^e^ | 4 | 0.40 [0.16, 1.03], I^2^%=69 | - | 0 | 0.19 [0.01, 4.14], I^2^% =NA | - | 1 | 2.93 [0.50, 17.11], I^2^%=0 |
| 5.5 | - | 7 | 0.44 [0.28, 0.70], I^2^%=16 | 0.23 [0.05, 1.05], I^2^%=39^f^ | 3 | 0.37 [0.21, 0.65], I^2^%=1 | - | 2 | 2.64 [0.59, 11.83], I^2^%=0 |
| 6.0 | - | 6 | 0.41 [0.28, 0.62], I^2^%=0 | - | 1 | 0.40 [0.05, 3.57], I^2^%=0 | - | 1 | 2.93 [0.50, 4.25], I^2^%=0 |

- = no meaningful difference; NA= not applicable

^a^after removing study by Okuda; ^b^after removing study by Wang; ^c^after removing study by Ueda; ^d^after removing study by Imhorf; ^e^after removing study by Brüggemann 2011; ^f^after removing study by Koselke

**Appendix 7 Quality appraisal of included studies (Table S12)**

**Table S12 Quality appraisal of included studies (indicators from Newcastle-Ottawa Scale of cohort studies)**

| **Reference** | **Outcome and cutoff value** | **Quality appraisal (indicators from New-Castle-Ottawa scale)** | | | | | | | | | **Total score** |
| --- | --- | --- | --- | --- | --- | --- | --- | --- | --- | --- | --- |
|  |  | 1 | 2 | 3 | 4 | 5A^a^ | 5B^b^ | 6 | 7^c^ | 8^d^ |  |
| VRC used for treat IFI. | | | | | | | | | | |  |
| Brüggemann 2011 | treatment success, 1.0 ml/L | * | * | * | * | * | - | * | * | * | 8 |
| Brüggemann 2011 | treatment success, 1.5 ml/L | * | * | * | * | * | - | * | * | * | 8 |
| Brüggemann 2011 | treatment success, 2.0 ml/L | * | * | * | * | * | - | * | * | * | 8 |
| Brüggemann 2011 | treatment success, 3.0 ml/L | * | * | * | * | * | - | * | * | * | 8 |
| Brüggemann 2011 | hepatotoxicity, 3.0 ml/L | * | * | * | NR | * | * | * | * | * | 8 |
| Brüggemann 2011 | hepatotoxicity, 4.0 ml/L | * | * | * | NR | * | * | * | * | * | 8 |
| Brüggemann 2011 | hepatotoxicity, 5.0 ml/L | * | * | * | NR | * | * | * | * | * | 8 |
| Brüggemann 2011 | hepatotoxicity, 5.5ml/L | * | * | * | NR | * | * | * | * | * | 8 |
| Brüggemann 2011 | hepatotoxicity, 6.0 ml/L | * | * | * | NR | * | * | * | * | * | 8 |
| Chu 2013 | treatment success, 1.0 ml/L | * | * | * | * | NR | * | * | * | * | 8 |
| Chu 2013 | hepatotoxicity, 5.5ml/L | * | * | * | NR | NR | * | * | * | * | 7 |
| Chu 2013 | neurotoxicity, 5.5ml/L | * | * | * | NR | NR | * | * | * | * | 7 |
| Chu 2013 | Visual disturbance, 5.5ml/L | * | * | * | NR | NR | * | * | * | * | 7 |
| Denning 2002 | treatment success, 0.5ml/L | * | * | * | * | NR | - | * | * | * | 7 |
| Denning 2002 | hepatotoxicity, 6.0 ml/L | * | * | * | * | NR | - | * | - | * | 6 |
| Gomez 2012 | treatment success,0.5 ml/L | * | * | * | * | * | * | * | * | * | 9 |
| Gomez 2012 | treatment success,1.0 ml/L | * | * | * | * | * | * | * | * | * | 9 |
| Gomez 2012 | treatment success,1.5 ml/L | * | * | * | * | * | * | * | * | * | 9 |
| Gomez 2012 | treatment success, 2.0 ml/L | * | * | * | * | * | * | * | * | * | 9 |
| Gomez 2012 | treatment success, 3.0 ml/L | * | * | * | * | * | * | * | * | * | 9 |
| Gomez 2012 | all cause mortality 0.35 ml/L | * | * | * | * | * | * | * | * | * | 9 |
| Gomez 2012 | treatment success,0.5 ml/L | * | * | * | * | * | * | * | * | * | 9 |
| Gomez 2012 | treatment success,1.0 ml/L | * | * | * | * | * | * | * | * | * | 9 |
| Gomez 2012 | treatment success,1.5 ml/L | * | * | * | * | * | * | * | * | * | 9 |
| Gomez 2012 | treatment success, 2.0 ml/L | * | * | * | * | * | * | * | * | * | 9 |
| Gomez 2012 | treatment success, 3.0 ml/L | * | * | * | * | * | * | * | * | * | 9 |
| Imhof 2006 | neurotoxicity, 3.0ml/L | * | * | * | * | NR | * | * | * | * | 8 |
| Imhof 2006 | neurotoxicity, 4.0ml/L | * | * | * | * | NR | * | * | * | * | 8 |
| Kim 2013 | treatment success,0.5 ml/L | * | * | * | * | NR | * | * | * | * | 8 |
| Kim 2013 | treatment success,1.0 ml/L | * | * | * | * | NR | * | * | * | * | 8 |
| Kim 2013 | treatment success,1.5 ml/L | * | * | * | * | NR | * | * | * | * | 8 |
| Kim 2013 | treatment success, 2.0 ml/L | * | * | * | * | NR | * | * | * | * | 8 |
| Kim 2013 | treatment success, 3.0 ml/L | * | * | * | * | NR | * | * | * | * | 8 |
| Kim 2011 | neurotoxicity, 6.0ml/L | * | * | * | NR | NR | * | * | * | * | 7 |
| Kim 2011 | hepatotoxicity,, 6.0ml/L | * | * | * | NR | NR | * | * | * | * | 7 |
| Koselke 2012 | neurotoxicity, 5.5 ml/L | * | * | * | NR | NR | * | * | NR | * | 6 |
| Koselke 2012 | hepatotoxicity,, 5.5 ml/L | * | * | * | NR | NR | * | * | NR | * | 6 |
| Lee 2013 | treatment success,0.5 ml/L | * | * | * | * | NR | - | * | * | * | 7 |
| Lee 2013 | treatment success,1.0 ml/L | * | * | * | * | NR | - | * | * | * | 7 |
| Lee 2013 | treatment success,2.0 ml/L | * | * | * | * | NR | - | * | * | * | 7 |
| Lee 2013 | treatment success, 3.0 ml/L | * | * | * | * | NR | - | * | * | * | 7 |
| Matsumoto 2009 | hepatotoxicity, 4.0 ml/L | NR | * | * | * | * | * | * | NR | NR | 6 |
| Okuda 2008 | treatment success,0.5 ml/L | * | * | * | * | * | - | * | NR | * | 7 |
| Okuda 2008 | treatment success,1.0 ml/L | * | * | * | * | * | - | * | NR | * | 7 |
| Okuda 2008 | treatment success,1.5 ml/L | * | * | * | * | * | - | * | NR | * | 7 |
| Okuda 2008 | treatment success, 2.0 ml/L | * | * | * | * | * | - | * | NR | * | 7 |
| Okuda 2008 | treatment success, 3.0 ml/L | * | * | * | * | * | - | * | NR | * | 7 |
| Okuda 2008 | hepatotoxicity, 3.0 ml/L | * | * | * | * | * | * | * | NR | * | 8 |
| Okuda 2008 | hepatotoxicity, 4.0 ml/L | * | * | * | * | * | * | * | NR | * | 8 |
| Okuda 2008 | hepatotoxicity, 5.0 ml/L | * | * | * | * | * | * | * | NR | * | 8 |
| Okuda 2008 | hepatotoxicity, 5.5ml/L | * | * | * | * | * | * | * | NR | * | 8 |
| Okuda 2008 | hepatotoxicity, 6.0 ml/L | * | * | * | * | * | * | * | NR | * | 8 |
| Okuda 2008 | neurotoxicity, 3.0 ml/L | * | * | * | * | * | * | * | NR | * | 8 |
| Okuda 2008 | neurotoxicity, 4.0 ml/L | * | * | * | * | * | * | * | NR | * | 8 |
| Okuda 2008 | neurotoxicity, 5.0 ml/L | * | * | * | * | * | * | * | NR | * | 8 |
| Okuda 2008 | neurotoxicity, 5.5ml/L | * | * | * | * | * | * | * | NR | * | 8 |
| Okuda 2008 | neurotoxicity, 6.0 ml/L | * | * | * | * | * | * | * | NR | * | 8 |
| Psacual 2008 | treatment success,1.0 ml/L | * | * | * | * | NR | - | * | NR | * | 6 |
| Psacual 2008 | hepatotoxicity, 5.5ml/L | * | * | * | NR | NR | * | * | * | * | 7 |
| Psacual 2008 | neurotoxicity, 5.5ml/L | * | * | * | NR | NR | * | * | * | * | 7 |
| Racil 2012 | treatment success,1.0 ml/L | * | * | * | * | NR | * | * | * | * | 8 |
| Racil 2012 | treatment success,2.0 ml/L | * | * | * | * | NR | * | * | * | * | 8 |
| Suzuki 2013 | hepatotoxicity, 4.0 ml/L | * | * | * | NR | NR | * | * | * | * | 7 |
| Wang 2014 | treatment success,0.5 ml/L | * | * | * | * | * | * | * | * | * | 9 |
| Wang 2014 | treatment success,1.0 ml/L | * | * | * | * | - | * | * | * | * | 8 |
| Wang 2014 | treatment success,1.5 ml/L | * | * | * | * | * | * | * | * | * | 9 |
| Wang 2014 | treatment success, 2.0 ml/L | * | * | * | * | * | * | * | * | * | 9 |
| Wang 2014 | treatment success, 3.0 ml/L | * | * | * | * | * | * | * | * | * | 9 |
| Wang 2014 | hepatotoxicity, 3.0 ml/L | * | * | * | NR | * | * | * | * | * | 8 |
| Wang 2014 | hepatotoxicity, 4.0 ml/L | * | * | * | NR | * | * | * | * | * | 8 |
| Wang 2014 | hepatotoxicity, 5.0 ml/L | * | * | * | NR | * | * | * | * | * | 8 |
| Wang 2014 | hepatotoxicity, 5.5ml/L | * | * | * | NR | * | * | * | * | * | 8 |
| Wang 2014 | hepatotoxicity, 6.0 ml/L | * | * | * | NR | * | * | * | * | * | 8 |
| Ueda 2009 | treatment success,0.5 ml/L | * | * | * | * | * | - | * | - | * | 7 |
| Ueda 2009 | treatment success,1.0 ml/L | * | * | * | * | * | - | * | - | * | 7 |
| Ueda 2009 | treatment success,1.5 ml/L | * | * | * | * | * | - | * | - | * | 7 |
| Ueda 2009 | treatment success, 2.0 ml/L | * | * | * | * | * | - | * | - | * | 7 |
| Ueda 2009 | treatment success, 3.0 ml/L | * | * | * | * | * | - | * | - | * | 7 |
| Ueda 2009 | hepatotoxicity, 3.0 ml/L | * | * | * | NR | * | * | * | * | * | 8 |
| Ueda 2009 | hepatotoxicity, 4.0 ml/L | * | * | * | NR | * | * | * | * | * | 8 |
| Ueda 2009 | hepatotoxicity, 5.0 ml/L | * | * | * | NR | * | * | * | * | * | 8 |
| Ueda 2009 | hepatotoxicity, 5.5ml/L | * | * | * | NR | * | * | * | * | * | 8 |
| Ueda 2009 | hepatotoxicity, 6.0 ml/L | * | * | * | NR | * | * | * | * | * | 8 |
| Gomez 2012 | treatment success,0.5 ml/L | * | * | * | * | * | * | * | - | * | 8 |
| Gomez 2012 | treatment success,1.0 ml/L | * | * | * | * | * | * | * | - | * | 8 |
| Gomez 2012 | treatment success,1.5 ml/L | * | * | * | * | * | * | * | - | * | 8 |
| Gomez 2012 | treatment success, 2.0 ml/L | * | * | * | * | * | * | * | - | * | 8 |
| Gomez 2012 | treatment success, 3.0 ml/L | * | * | * | * | * | * | * | - | * | 8 |
| VRC used for prevent from fungal disease | | | | | | | | | | |  |
| Mitsani 2012 | incidence of IFI, 1.0 ml/L | * | * | * | * | NR | * | * | * | * | 8 |
| Mitsani 2012 | incidence of IFI, 1.5ml/L | * | * | * | * | NR | * | * | * | * | 8 |
| Heng 2013 | incidence of IFI, 0.5 ml/L | * | * | * | NR | * | * | * | * | * | 8 |
| Heng 2013 | incidence of IFI, 1.0 ml/L | * | * | * | NR | * | * | * | * | * | 8 |
| Heng 2013 | incidence of IFI, 1.5 ml/L | * | * | * | NR | * | * | * | * | * | 8 |
| Heng 2013 | incidence of IFI, 2.0 ml/L | * | * | * | NR | * | * | * | * | * | 8 |
| Heng 2013 | incidence of IFI, 3.0 ml/L | * | * | * | NR | * | * | * | * | * | 8 |
| Brüggemann 2010 | incidence of IFI, 0.5 ml/L | * | * | * | * | * | * | * | - | * | 8 |
| Brüggemann 2010 | incidence of IFI, 1.0 ml/L | * | * | * | * | * | * | * | - | * | 8 |
| Brüggemann 2010 | incidence of IFI, 1.5 ml/L | * | * | * | * | * | * | * | - | * | 8 |
| Brüggemann 2010 | incidence of IFI, 2.0 ml/L | * | * | * | * | * | * | * | - | * | 8 |
| Brüggemann 2010 | incidence of IFI, 3.0 ml/L | * | * | * | * | * | * | * | - | * | 8 |
| Brüggemann 2010 | Visual disturbance, 3.0 ml/L | * | * | * | NR | * | * | * | * | * | 8 |
| Brüggemann 2010 | Visual disturbance, 4.0 ml/L | * | * | * | NR | * | * | * | * | * | 8 |
| Brüggemann 2010 | Visual disturbance, 5.0 ml/L | * | * | * | NR | * | * | * | * | * | 8 |
| Brüggemann 2010 | Visual disturbance, 5.5 ml/L | * | * | * | NR | * | * | * | * | * | 8 |
| Brüggemann 2010 | Visual disturbance, 6.0 ml/L | * | * | * | NR | * | * | * | * | * | 8 |
| Trifilio 2007 | incidence of IFI, 0.5 ml/L | * | * | * | - | NR | * | * | * | * | 7 |
| Trifilio 2007 | incidence of IFI, 1.0 ml/L | * | * | * | - | NR | * | * | * | * | 7 |
| Trifilio 2007 | incidence of IFI, 2.0 ml/L | * | * | * | - | NR | * | * | * | * | 7 |
| Trifilio 2007 | IFI related mortality, 0.5mg/L | * | * | * | * | NR | * | * | * | * | 8 |
| Trifilio 2007 | IFI related mortality, 1.0 ml/L | * | * | * | * | NR | * | * | * | * | 8 |
| Trifilio 2007 | IFI related mortality, 2.0 ml/L | * | * | * | * | NR | * | * | * | * | 8 |
| Mori 2015 | Visual disturbance, 3.0 ml/L | * | * | * | NR | * | * | * | * | * | 8 |
| Mori 2015 | Visual disturbance, 4.0 ml/L | * | * | * | NR | * | * | * | * | * | 8 |
| Mori 2015 | Visual disturbance, 5.0 ml/L | * | * | * | NR | * | * | * | * | * | 8 |
| Mori 2015 | Visual disturbance, 5.5 ml/L | * | * | * | NR | * | * | * | * | * | 8 |
| Mori 2015 | Visual disturbance, 6.0 ml/L | * | * | * | NR | * | * | * | * | * | 8 |
| Mori 2015 | hepatotoxicity, 3.0 ml/L | * | * | * | * | * | * | * | * | * | 9 |
| Mori 2015 | hepatotoxicity, 4.0 ml/L | * | * | * | * | * | * | * | * | * | 9 |
| Mori 2015 | hepatotoxicity, 5.0 ml/L | * | * | * | * | * | * | * | * | * | 9 |
| Mori 2015 | hepatotoxicity, 5.5ml/L | * | * | * | * | * | * | * | * | * | 9 |
| Mori 2015 | hepatotoxicity, 6.0 ml/L | * | * | * | * | * | * | * | * | * | 9 |

VRC= voriconazole; IFI= invasive fungal infection; NA=not application; NR= not reported

^a^Studies that reported age clearly received one score

^b^ When evaluating efficacy, studies that clearly reported category of IFI received one score if VRC used for treatment, studies clearly reported type of transplant received one score if VRC used for prophylaxis; when evaluating safety, studies that clearly reported population received one score.

^c^ Studies with follow-up time >4 weeks was assigned one score when evaluated efficacy, studies with follow-up time >1 week was assigned one score when evaluated safety.

^d^ Studies that reported a follow-up rate was assigned one score.

1. Indicates exposed cohort truly representative.

2. Non-exposed cohort drawn from a same source.

3. Ascertainment of exposure from a secure record.

4. Outcome of interest not present at start of study.

5A. Cohorts comparable on basis of main factor

5B. Cohorts comparable on other factors

6. Assessment of outcome of record linkage or independent blind assessment.

7. Follow-up long enough for outcomes to occur.

8. Complete accounting for cohorts

**Appendix 8 Forest plot for different outcomes at each cutoff value (Figure S1-S55)**

**Appendix 8.1 Forest plot for rate of treatment success at each cutoff value (Figure S1-S4)**

**Figure S1 Meta-analysis for rate of treatment success** **(trough concentration of <1.0 mg/L comparison with >1.0 mg/L, RR <1 favours C_trough_ >1.0 mg/L)**

**Figure S2 Meta-analysis for rate of treatment success (trough concentration of <1.5 mg/L comparison with >1.5 mg/L, RR <1 favours C_trough_ >1.5 mg/L)**

**Figure S3 Meta-analysis for rate of treatment (trough concentration of <2.0 mg/L comparison with >2.0 mg/L, RR <1 favours C_trough_ >2.0 mg/L)**

**Figure S4 Meta-analysis for rate of treatment success (trough concentration of <3.0 mg/L comparison with >3.0 mg/L, RR <1 favours C_trough_ >3.0 mg/L)**

**Appendix 8.2 forest plot for subgroup analysis of rate of treatment success at each cutoff value (Figure S5-S19)**

**Figure S5 Subgroup analysis for rate of treatment success stratified by category of diagnosis (trough concentration of <0.5mg/L comparison with >0.5mg/L, RR <1 favours C_trough_ >0.5 mg/L)**

**Figure S6 Subgroup analysis for rate of treatment success stratified by adults or children (trough concentration of <0.5mg/L comparison with >0.5mg/L, RR <1 favours C_trough_ >0.5 mg/L)**

**Figure S7 Subgroup analysis for rate of treatment success stratified by monotherapy or combo therapy (trough concentration of <0.5mg/L comparison with >0.5mg/L, RR <1 favours C_trough_ >0.5 mg/L)**

**Figure S8 Subgroup analysis for rate of treatment success stratified by category of diagnosis (trough concentration of <1.0 mg/L comparison with >1.0 mg/L, RR <1 favours C_trough_ >1.0 mg/L)**

**Figure S9 Subgroup analysis for rate of treatment success stratified by adults or children (trough concentration of <1.0 mg/L comparison with >1.0 mg/L, RR <1 favours C_trough_ >1.0 mg/L)**

**Figure S10 Subgroup analysis for rate of treatment success stratified by monotherapy or combo therapy (trough concentration of <1.0 mg/L comparison with >1.0mg/L, RR <1 favours C_trough_ >1.0 mg/L)**

**Figure S11 Subgroup analysis for rate of treatment success stratified by category of diagnosis (trough concentration of <1.5 mg/L comparison with >1.5 mg/L, RR <1 favours C_trough_ >1.5 mg/L)**

**Figure S12 Subgroup analysis for rate of treatment success stratified by adults or children (trough concentration of <1.5 mg/L comparison with >1.5 mg/L, RR <1 favours C_trough_ >1.5 mg/L)**

**Figure S13 Subgroup analysis for rate of treatment success stratified by monotherapy or combo therapy (trough concentration of <1.5 mg/L comparison with >1.5 mg/L, RR <1 favours C_trough_ >1.5 mg/L)**

**Figure S14 Subgroup analysis for rate of treatment success stratified by category of diagnosis (trough concentration of <2.0 mg/L comparison with >2.0 mg/L, RR <1 favours C_trough_ >2.0 mg/L)**

**Figure S15 Subgroup analysis for rate of treatment success stratified by adults or children (trough concentration of <2.0 mg/L comparison with >2.0 mg/L, RR <1 favours C_trough_ >2.0 mg/L)**

**Figure S16 Subgroup analysis for rate of treatment success stratified by monotherapy or combo therapy (trough concentration of <2.0 mg/L comparison with >2.0 mg/L, RR <1 favours C_trough_ >2.0 mg/L)**

**Figure S17 Subgroup analysis for rate of treatment success stratified by category of diagnosis (trough concentration of <3.0 mg/L comparison with >3.0 mg/L, RR <1 favours C_trough_ >3.0 mg/L)**

**Figure S18 Subgroup analysis for rate of treatment success stratified by adults or children (trough concentration of <3.0 mg/L comparison with >3.0 mg/L, RR <1 favours C_trough_ >3.0 mg/L)**

**Figure S19 Subgroup analysis for rate of treatment success stratified by monotherapy or combo therapy (trough concentration of <3.0 mg/L comparison with >3.0 mg/L, RR <1 favours C_trough_ >3.0 mg/L)**

**Appendix 8.3 Forest plot for sensitivety analysis of treatment success at each cutoff value (Figure S20-S23)**

**Figure S20 Sensitivity analysis that excluded combo therapy based on individual data for rate of treatment success (trough concentration of <1.0 mg/L comparison with >1.0 mg/L, RR <1 favours C_trough_ >1.0 mg/L)**

**Figure S21 Sensitivity analysis that excluded combo therapy based on individual data for rate of treatment success (trough concentration of <1.5 mg/L comparison with >1.5 mg/L, RR <1 favours C_trough_ >1.5 mg/L)**

**Figure S22 Sensitivity analysis that excluded combo therapy based on individual data for rate of treatment (trough concentration of <2.0 mg/L comparison with >2.0 mg/L, RR <1 favours C_trough_ >2.0 mg/L)**

**Figure S23 Sensitivity analysis that excluded combo therapy based on individual data for rate of treatment success (trough concentration of <3.0 mg/L comparison with >3.0 mg/L, RR <1 favours C_trough_ >3.0 mg/L)**

**Appendix 8.4 Forest plot for all cause mortality at each cutoff value (Figure S24-S27)**

**Figure S24 Meta-analysis for all cause mortality (trough concentration of <0.5mg/L comparison with >0.5mg/L, RR <1 favours C_trough_ <0.5 mg/L)**

**Figure S25 Meta-analysis for all cause mortality (trough concentration of <1.0 mg/L comparison with >1.0 mg/L, RR <1 favours C_trough_ <1.0 mg/L)**

**Figure S26 Meta-analysis for all cause mortality (trough concentration of <1.5 mg/L comparison with >1.5 mg/L,** **RR <1 favours C_trough_ <1.5 mg/L)**

**Figure S27 Meta-analysis for all cause mortality (trough concentration of <2.0 mg/L comparison with >2.0 mg/L,** **RR <1 favours C_trough_ <2.0 mg/L)**

**Appendix 8.5 Forest plot for incidence of IFI at each cutoff value (Figure S28-S32)**

**Figure S28 Meta-analysis for incidence of IFI (trough concentration of <0.5mg/L comparison with >0.5mg/L,** **RR <1 favours C_trough_ <0.5 mg/L)**

**Figure S29 Meta-analysis for incidence of IFI (trough concentration of <1.0 mg/L comparison with >1.0 mg/L,** **RR <1 favours C_trough_ <1.0 mg/L)**

**Figure S30 Meta-analysis for incidence of IFI (trough concentration of <1.5 mg/L comparison with >1.5 mg/L,** **RR <1 favours C_trough_ <1.5 mg/L)**

**Figure S31 Meta-analysis for incidence of IFI (trough concentration of <2.0 mg/L comparison with >2.0 mg/L,** **RR <1 favours C_trough_ <2.0 mg/L)**

**Figure S32 Meta-analysis for incidence of IFI (trough concentration of <3.0 mg/L comparison with >3.0 mg/L,** **RR <1 favours C_trough_ <3.0 mg/L)**

**Appendix 8.6 Forest plot for safety at each cutoff value (Figure S33-S55)**

**Figure S33** **Meta-analysis for incidence of neurotoxicity (trough concentration of <3.0 mg/L comparison with >3.0 mg/L,** **RR <1 favours C_trough_ <3.0 mg/L)**

**Figure S34 Meta-analysis for incidence of visual disorder (trough concentration of <3.0 mg/L comparison with >3.0 mg/L,** **RR <1 favours C_trough_ <3.0 mg/L)**

**Figure S35** **Subgroup analysis for incidence of hepatotoxicity stratified by Asian and non-Asian (trough concentration of <3.0 mg/L comparison with >3.0 mg/L,** **RR <1 favours C_trough_ <3.0 mg/L)**

**Figure S36 Subgroup analysis for incidence of hepatotoxicity stratified by adults and children (trough concentration of <3.0 mg/L comparison with >3.0 mg/L,** **RR <1 favours C_trough_ <3.0 mg/L)**

**Figure S37 Meta-analysis for incidence of hepatotoxicity (trough concentration of <4.0 mg/L comparison with >4.0 mg/L, RR <1 favours C_trough_ <4.0 mg/L)**

**Figure S38 Meta-analysis for incidence of neurotoxicity (trough concentration of <4.0 mg/L comparison with >4.0 mg/L, RR <1 favours C_trough_ <4.0 mg/L)**

**Figure S39 Meta-analysis for incidence of visual disorder (trough concentration of <4.0 mg/L comparison with >4.0 mg/L, RR <1 favours C_trough_ <4.0 mg/L)**

**Figure S40 Subgroup analysis for incidence of hepatotoxicity stratified by Asian and non-Asian (trough concentration of <4.0 mg/L comparison with >4.0 mg/L, RR <1 favours C_trough_ <4.0 mg/L)**

**Figure S41 Subgroup analysis for incidence of hepatotoxicity stratified by adult and children (trough concentration of <4.0 mg/L comparison with >4.0 mg/L, RR <1 favours C_trough_ <4.0 mg/L)**

**Figure S42 Meta-analysis for incidence of hepatotoxicity (trough concentration of <5.0 mg/L comparison with >5.0 mg/L, RR <1 favours C_trough_ <5.0 mg/L)**

**Figure S43 Meta-analysis for incidence of visual disorder (trough concentration of <5.0 mg/L comparison with >5.0 mg/L, RR <1 favours C_trough_ <5.0 mg/L)**

**Figure S44 Subgroup analysis for incidence of hepatotoxicity stratified by Asian and non-Asian (trough concentration of <5.0 mg/L comparison with >5.0 mg/L, RR <1 favours C_trough_ <5.0 mg/L)**

**Figure S45 Subgroup analysis for incidence of hepatotoxicity stratified by adult and children (trough concentration of <5.0 mg/L comparison with >5.0 mg/L, RR <1 favours C_trough_ <5.0 mg/L)**

**Figure S46 Meta-analysis for incidence of hepatotoxicity (trough concentration of <5.5 mg/L comparison with >5.5 mg/L, RR <1 favours C_trough_ <5.5 mg/L)**

**Figure S47 Meta-analysis for incidence of neurotoxicity (trough concentration of <5.5 mg/L comparison with >5.5 mg/L, RR <1 favours C_trough_ <5.5 mg/L)**

**Figure S48 Meta-analysis for incidence of visual disorder (trough concentration of <5.5 mg/L comparison with >5.5 mg/L, RR <1 favours C_trough_ <5.5 mg/L)**

**Figure S49 Subgroup analysis for incidence of hepatotoxicity stratified by Asian and non-Asian (trough concentration of <5.5 mg/L comparison with >5.5 mg/L, RR <1 favours C_trough_ <5.5 mg/L)**

**Figure S50 Subgroup analysis for incidence of hepatotoxicity stratified by adult and children (trough concentration of <5.5 mg/L comparison with >5.5 mg/L, RR <1 favours C_trough_ <5.5 mg/L)**

**Figure S51 Meta-analysis for incidence of hepatotoxicity (trough concentration of <6.0 mg/L comparison with >6.0mg/L, RR <1 favours C_trough_ <6.0 mg/L)**

**Figure S52 Meta-analysis for incidence of neurotoxicity (trough concentration of <6.0 mg/L comparison with >6.0mg/L, RR <1 favours C_trough_ <6.0 mg/L)**

**Figure S53 Meta-analysis for incidence of visual disorder (trough concentration of <6.0 mg/L comparison with >6.0mg/L, RR <1 favours C_trough_ <6.0 mg/L)**

**Figure S54 Subgroup analysis for incidence of hepatotoxicity stratified by Asian and non-Asian (trough concentration of <6.0 mg/L comparison with >6.0mg/L, RR <1 favours C_trough_ <6.0 mg/L)**

**Figure S55 Subgroup analysis for incidence of hepatotoxicity stratified by adult and children (trough concentration of <6.0 mg/L comparison with >6.0mg/L, RR <1 favours C_trough_ <6.0 mg/L)**
